# Alzheimer disease pathology and the cerebrospinal fluid proteome

**DOI:** 10.1186/s13195-018-0397-4

**Published:** 2018-07-18

**Authors:** Loïc Dayon, Antonio Núñez Galindo, Jérôme Wojcik, Ornella Cominetti, John Corthésy, Aikaterini Oikonomidi, Hugues Henry, Martin Kussmann, Eugenia Migliavacca, India Severin, Gene L. Bowman, Julius Popp

**Affiliations:** 1 Nestlé Institute of Health Sciences, Lausanne, Switzerland; 2 Precision for Medicine, Geneva, Switzerland; 3 Old Age Psychiatry, Department of Psychiatry, CHUV, Lausanne, Switzerland; 4 Department of Laboratories, CHUV, Lausanne, Switzerland; 5 Geriatric Psychiatry, Department of Mental Health and Psychiatry, HUG, Geneva, Switzerland; 6 Present address: Liggins Institute, University of Auckland, Auckland, New Zealand

**Keywords:** Alzheimer disease, Amyloid, Biomarker, Cerebrospinal fluid, CSF, Mass spectrometry, Proteomics, Tau, Tandem mass tag

## Abstract

**Background:**

Altered proteome profiles have been reported in both postmortem brain tissues and body fluids of subjects with Alzheimer disease (AD), but their broad relationships with AD pathology, amyloid pathology, and tau-related neurodegeneration have not yet been fully explored. Using a robust automated MS-based proteomic biomarker discovery workflow, we measured cerebrospinal fluid (CSF) proteomes to explore their association with well-established markers of core AD pathology.

**Methods:**

Cross-sectional analysis was performed on CSF collected from 120 older community-dwelling adults with normal (*n* = 48) or impaired cognition (*n* = 72). LC-MS quantified hundreds of proteins in the CSF. CSF concentrations of β-amyloid 1–42 (Aβ_1–42_), tau, and tau phosphorylated at threonine 181 (P-tau181) were determined with immunoassays. First, we explored proteins relevant to biomarker-defined AD. Then, correlation analysis of CSF proteins with CSF markers of amyloid pathology, neuronal injury, and tau hyperphosphorylation (i.e., Aβ_1–42_, tau, P-tau181) was performed using Pearson’s correlation coefficient and Bonferroni correction for multiple comparisons.

**Results:**

We quantified 790 proteins in CSF samples with MS. Four CSF proteins showed an association with CSF Aβ_1–42_ levels (*p* value ≤ 0.05 with correlation coefficient (*R*) ≥ 0.38). We identified 50 additional CSF proteins associated with CSF tau and 46 proteins associated with CSF P-tau181 (*p* value ≤ 0.05 with *R* ≥ 0.37). The majority of those proteins that showed such associations were brain-enriched proteins. Gene Ontology annotation revealed an enrichment for synaptic proteins and proteins originating from reelin-producing cells and the myelin sheath.

**Conclusions:**

We used an MS-based proteomic workflow to profile the CSF proteome in relation to cerebral AD pathology. We report strong evidence of previously reported CSF proteins and several novel CSF proteins specifically associated with amyloid pathology or neuronal injury and tau hyperphosphorylation.

**Electronic supplementary material:**

The online version of this article (10.1186/s13195-018-0397-4) contains supplementary material, which is available to authorized users.

## Background

Proteome alterations have been identified in a multitude of pathologies, such as cancer, metabolic disorders, and brain diseases [1]. Several circulating protein markers of neurodegenerative diseases, such as Parkinson’s disease or Alzheimer disease (AD), have been reported [2], but the ones with consistent findings or of current clinical utility are very few [3]. AD is the most common form of dementia, and there is still an urgent need for the definition of early detection markers as well as for a better understanding of its pathogenesis. In the latter perspective, cerebrospinal fluid (CSF) represents a key biofluid to decipher altered protein levels and pathways in diseases of the central nervous system (CNS) using large-scale proteomic technologies, such as MS-based platforms.

Because of the proximity of CSF to the brain and the presence of proteins in CSF specific to the brain [4, 5], the CSF proteome can reflect the biochemical and metabolic changes in the CNS. In particular, despite the definitive confirmation of the diagnosis of AD being possible today only at brain autopsy, specific CSF peptides and proteins (i.e., β-amyloid 1–42 [Aβ_1–42_], total tau, and hyperphosphorylated tau [P-tau]) linked to the main hallmarks of AD pathology, such as amyloid plaques and neurofibrillary tangles, can complement clinical examination for the diagnosis of AD [6, 7].

There is now strong evidence that suggests the development of AD pathology begins years to decades prior to the onset of the first clinical signs. Thus, on one hand, elderly persons with normal cognition may already have cerebral AD pathology and be at the preclinical stage of the disease [8]; on the other hand, subjects with cognitive deficits may present with cognitive impairment suggesting AD but not primarily or only partially related to AD pathology. New research criteria consider AD as a biological *continuum* across the clinical spectrum from asymptomatic stage to advanced dementia and emphasize the utility of biomarkers of AD pathology for an accurate diagnosis, in particular at the preclinical and prodromal disease stages [8–10]. In this respect, endophenotype approaches have been proposed as innovative ways to better address AD stages using proxy measures such as the concentrations of the aforementioned CSF markers of core AD pathology [11].

Several studies have characterized the CSF proteome with MS but mainly using sample pools and/or a limited number of samples [12–14]. Because of technical constraints such as limited sample throughput [15], studies in larger clinical cohorts using MS-based proteomics are indeed limited [16–21]. In recent years, our group [22] and other groups [23, 24] have demonstrated that MS-based proteomics enables protein biomarker discovery in large numbers of human clinical samples, providing increased statistical power and result robustness [21, 22, 25]. Although most of these studies were performed with plasma or serum samples [26], the analysis of the CSF proteome and its alteration using MS-based proteomics in larger cohorts has been mostly unexplored.

**Fig. 1:**
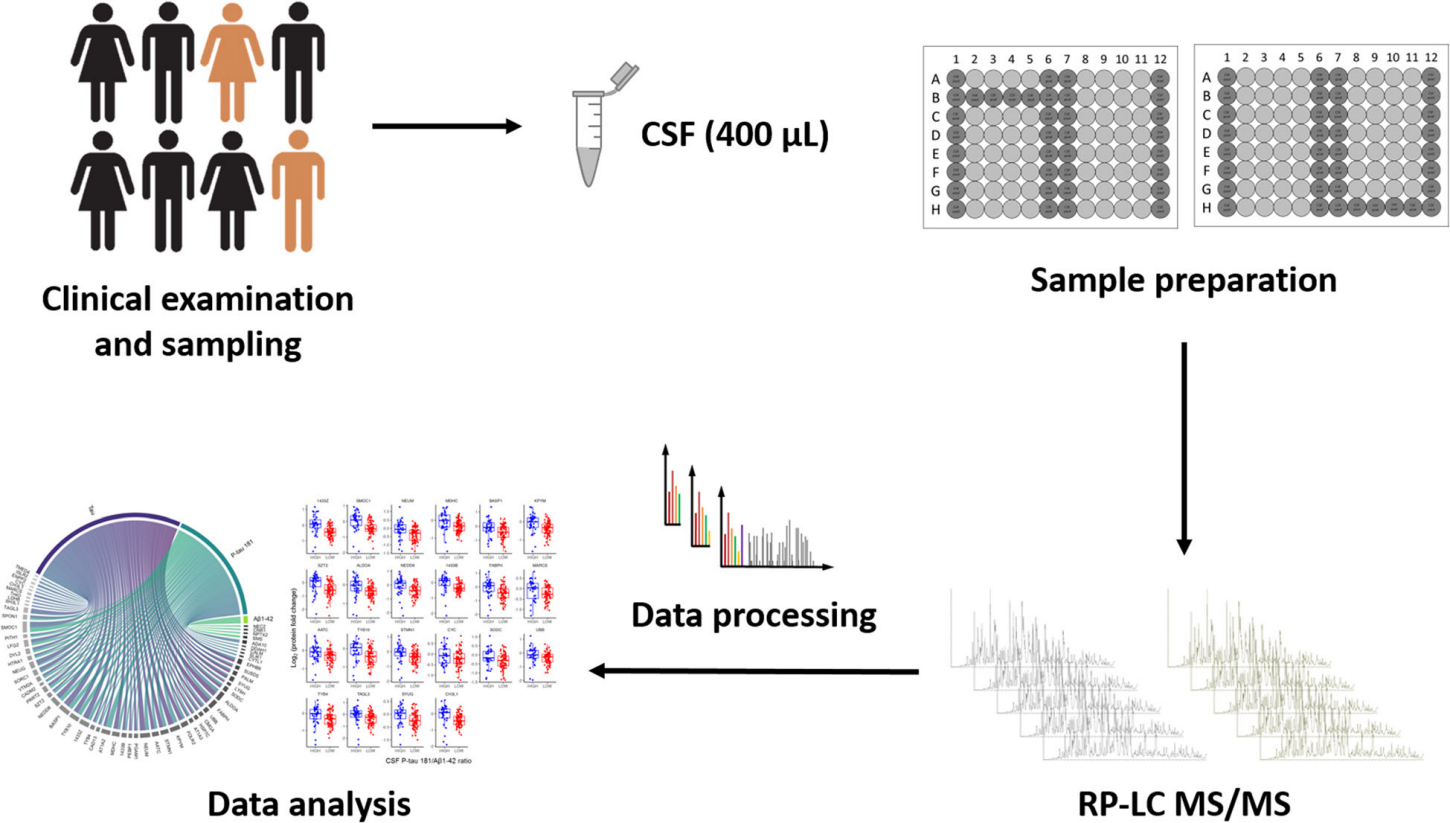
Study design and cerebrospinal fluid (CSF) proteome profiling workflow. CSF samples from 120 older individuals with or without cognitive impairment were analyzed using a highly automated shotgun MS-based proteomic workflow. The workflow consists of first removing 14 highly abundant proteins in CSF by immunoaffinity. The rest of the workflow is automated in a 96-well plate format and includes steps of (1) reduction, alkylation, and enzymatic digestion; (2) isobaric labeling and pooling; and (3) purifications. The samples are analyzed with reversed-phase LC-MS/MS, and the data are processed with standard bioinformatic tools

Our aim in this study was to investigate the CSF proteome in relation to the core elements of CSF-defined AD pathology in older adults (*n* = 120) with normal and impaired cognition using MS-based shotgun proteomics (Fig. 1). We evaluated whether the CSF proteome could relate to AD pathology, defined as the combined presence of both amyloid pathology and tau pathology. We then explored more deeply the relationships of the quantified proteins in CSF with well-established biomarkers of amyloid pathology, neuronal injury, and tau hyperphosphorylation (i.e., Aβ_1–42_, tau, and tau phosphorylated at threonine 181 [P-tau181], respectively).

## Methods

### Study design

One hundred twenty community-dwelling participants were included in this study, of whom 48 were cognitively healthy volunteers and 72 had mild cognitive impairment (MCI) (*n* = 63) or mild dementia of AD type (*n* = 9) [27]. Diagnosis of MCI or dementia was based on neuropsychological and clinical evaluation and made by a consensus conference of psychiatrists and/or neurologists as well as neuropsychologists prior to inclusion in the study. The participants with cognitive impairment were recruited from among outpatients who were referred to the Memory Clinics, Departments of Psychiatry, and Department of Clinical Neurosciences, University Hospitals of Lausanne, Switzerland. They had no major psychiatric disorders or substance abuse or severe or unstable physical illness that might contribute to cognitive impairment, had a Clinical Dementia Rating (CDR) [28] score > 0, and met the clinical diagnostic criteria for MCI [29] or AD mild dementia according to the recommendations of the National Institute on Aging-Alzheimer’s Association [30]. In the current study, nine subjects met criteria for probable AD dementia. Because there is a clinical *continuum* between MCI and mild dementia, and because the participants with cognitive impairment were patients from memory clinics recruited in the same way regardless of MCI or mild dementia classification, these subjects were grouped and labeled as cognitively impaired with CDR > 0 (Table 1). The control subjects were recruited through journal announcements or word of mouth and had no history, symptoms, or signs of relevant psychiatric or neurologic disease and no cognitive impairment (CDR = 0). All participants underwent a comprehensive clinical and neuropsychological evaluation, structural brain imaging, and venous and lumbar punctures [27]. Magnetic resonance imaging (MRI) and computed tomographic scans were used to exclude cerebral pathologies possibly interfering with cognitive performance.

**Table 1:** Demographics and clinical characteristics

	P-tau181/Aβ_1–42_ ≤ 0.0779 (*n* = 78)	P-tau181/Aβ_1–42_ > 0.0779 (*n* = 42)	CDR = 0 (*n* = 48)	CDR > 0 (*n* = 72)
Age, yr, mean (SD)	68.4 (8.3)	74.1 (5.6)^a^	66.0 (7.4)	73.3 (6.9)^a^
Gender, *n* (%) of males	25 (32.05%)	18 (42.86%)	17 (35.42%)	26 (36.11%)
Education, yr, mean (SD)	12.5 (2.7)	12.1 (2.4)	13.2 (2.3)	11.8 (2.7)^a^
CDR, score (% of subjects, number of subjects)	0 (60.2%, 47) or 0.5 (37.2%, 29) or 1 (2.6%, 2)	0 (2.4%, 1) or 0.5 (80.9%, 34) or 1 (16.7%, 7)	0 (100%, 48)	0.5 (87.5%, 63) or 1 (12.5%, 9)
MMSE score, mean (SD)	27.8 (2.3)	25.2 (3.7)^a^	28.5 (1.4)	25.9 (3.5)^a^
*APOE* ε4 carriers, *n* (%)	13 (16.67%)	24 (57.14%)^a^	11 (22.92%)	26 (36.11%)^a^
CSF Aβ_1–42_ (pg/ml), mean (SD)	979.9 (196.4)	601.2 (190.0)^a^	957.4 (194.0)	774.0 (281.5)^a^
CSF tau (pg/ml), mean (SD)	235.1 (104.2)	624.2 (322.4)^a^	221.5 (82.9)	471.1 (316.6)^a^
CSF P-tau181 (pg/ml), mean (SD)	46.7 (13.4)	90.3 (44.8)^a^	45.9 (13.3)	72.7 (40.9)^a^
CSF P-tau181/Aβ_1–42_, mean (SD)	0.05 (0.01)	0.16 (0.10)^a^	0.049 (0.015)	0.114 (0.097)^a^
CSF albumin index^b^, mean (SD)	5.9 (2.4)	6.4 (2.3)	5.3 (1.9)	6.6 (2.5)^a^

*Abbreviations: Aβ*_*1–42*_ β-Amyloid 1–42, *APOE* Apolipoprotein E, *CDR* Clinical Dementia Rating, *CSF* Cerebrospinal fluid, *MMSE* Mini Mental State Examination, *P-tau181* Tau phosphorylated at threonine 181

^a^Statistically different (*p* ≤ 0.05) from P-tau181/Aβ_1–42_ ≤ 0.0779, and CDR = 0, respectively, using *t* tests for continuous variables and binomial proportion tests for categorical variables. ^b^CSF albumin index = [CSF albumin]/[serum albumin] × 100

Neuropsychological tests were used to assess cognitive performance in the domains of memory [31], language, and visuoconstructive functions. The Mini Mental State Examination [32] was used to assess participants’ global cognitive performance. Depression and anxiety were assessed using the Hospital Anxiety and Depression Scale [33]. The psychosocial and functional assessments included activities of daily living and instrumental activities of daily living, the Neuropsychiatric Inventory Questionnaire, and the Informant Questionnaire on Cognitive Decline in the Elderly [34], and these were completed by family members of the participants. All tests and scales are validated and widely used in the field.

### CSF sample collection

Lumbar punctures were performed between 8:30 a.m. and 9:30 a.m. after overnight fasting. A standardized technique with a 22-gauge “atraumatic” spinal needle and a sitting or lying position was applied [35]. A volume of 10–12 ml of CSF was collected in polypropylene tubes. Routine cell count and protein quantification were performed. The remaining CSF was frozen in aliquots (500 μl) no later than 1 hour after collection and stored at − 80 °C without thawing until experiment and assay.

### MS-based proteomics

CSF samples were prepared using a highly automated shotgun proteomic workflow as previously described [36] and isobaric tags [37] for relative quantification of proteins. Reversed-phase LC-MS/MS was performed with a hybrid linear ion trap-Orbitrap Elite and an UltiMate 3000 RSLCnano System (Thermo Scientific, Waltham, MA, USA) as recently described [38]. Protein identification was performed against the human UniProtKB/Swiss-Prot database (08/12/2014 release). All details are provided in Additional file 1: Supplementary Methods.

### CSF β-amyloid 1–42, tau, tau phosphorylated at threonine 181, and *APOE* genotyping

The measurements were performed using commercially available enzyme-linked immunosorbent assay kits and TaqMan assays as described in Additional file 1: Supplementary Methods.

### Definition of CSF biomarker profile of Alzheimer pathology

A pathological AD CSF biomarker profile was defined as CSF P-tau181/Aβ_1–42_ ratio > 0.0779 (i.e., “high” ratio for positive CSF profile of AD pathology), based on clinical study site data [39] and in line with previous work (i.e., 0.08) [40]. The cutoff optimized the Youden index [41] of the ROC curve for the prediction of CDR categories (CDR = 0 versus CDR > 0) as previously reported [27], where the cutoff for CSF P-tau181/Aβ_1–42_ ratio was further confirmed to be a highly significant predictor of cognitive decline.

#### Proteomic data management

Six CSF samples were removed because of aberrant values, leaving CSF proteomic data available for 114 subjects (exclusion of those 6 subjects did not induce bias on the overall population characteristics) (*see* Additional file 1: Table S1)). In total, 790 CSF proteins were quantified.

For exploration of CSF proteins relevant to AD pathology (*see below*), proteins with > 5% missingness were excluded, leaving 541 CSF proteins. The remaining missing data (5% or less per protein) were imputed by randomly drawing a value between the observed range of biomarker values. Log_2_ of the protein ratio fold changes were scaled to mean zero and SD of 1 prior to statistical analyses. Calculation and statistics were performed with the R version 3.3.2 statistical software (http://www.r-project.org/).

### Exploratory analysis of CSF proteins relevant to Alzheimer pathology

In a first exploratory analysis, 541 CSF proteins were tested (one by one) in a logistic regression model as follows:

$$ \mathrm{Positive}\ \mathrm{CSF}\ \mathrm{profile}\ \mathrm{of}\ \mathrm{AD}\sim \mathrm{CSF}\ \mathrm{protein}\ \mathrm{biomarkers}+\mathrm{age}+\mathrm{gender}+\mathrm{years}\ \mathrm{of}\ \mathrm{education}+\mathrm{presence}\ \mathrm{of} APOE\ \upvarepsilon 4\ \mathrm{allele} $$

where positive CSF profile of AD is defined by categorizing the CSF P-tau181/Aβ_1–42_ ratio into two groups: P-tau181/Aβ_1–42_ > 0.0779 for AD CSF biomarker profile (or “high”) and P-tau181/Aβ_1–42_ ≤ 0.0779 for non-AD CSF biomarker profile (or “low”). *p* Values were corrected for multiple testing using the Benjamini-Hochberg procedure. Box plots were produced for the significant hits presenting false discovery rate (FDR) ≤ 5%.

### Selection of CSF proteins relevant to Alzheimer pathology

Least absolute shrinkage and selection operator (LASSO) logistic regression [42] selected biomarkers that best predict CSF biomarker profile of AD pathology. A reference model was initially generated, testing variables that are likely to be available to clinicians and known risk factors for AD to provide a benchmark for comparison with the model that included CSF proteins. These inputs included age, gender, years of education, and presence of the apolipoprotein E (*APOE*) ε4 allele, such as:

$$ \mathrm{Positive}\ \mathrm{CSF}\ \mathrm{profile}\ \mathrm{of}\ \mathrm{AD}\sim \mathrm{age}+\mathrm{gender}+\mathrm{years}\ \mathrm{of}\ \mathrm{education}+\mathrm{presence}\ \mathrm{of} APOE\ \upvarepsilon 4\ \mathrm{allele} $$

In addition to all variables used to make the reference models, CSF protein measurements (i.e., 541 CSF proteins) and CSF albumin index were then included in building so-called best models:

$$ \mathrm{Positive}\ \mathrm{CSF}\ \mathrm{profile}\ \mathrm{of}\ \mathrm{AD}\sim \mathrm{CSF}\ \mathrm{protein}\ \mathrm{biomarkers}+\mathrm{CSF}\ \mathrm{albumin}\ \mathrm{index}+\mathrm{age}+\mathrm{gender}+\mathrm{years}\ \mathrm{of}\ \mathrm{education}+\mathrm{presence}\ \mathrm{of} APOE\ \upvarepsilon 4\ \mathrm{allele} $$

A tenfold cross-validation process was performed for each LASSO analysis using the glmnet package [43], which allows estimating the confidence interval of the misclassification error for each value of the regularization parameter λ. The LASSO analyses were repeated 100 times (1000 times for the reference models). The model that minimized the upper limit of the cross-validated misclassification error confidence interval across the 100 runs with less than 20 features (when possible) was selected. The results were formally tested for significance against the reference model using accuracy with a McNemar test. The group differences for the CSF proteins selected in the best models were graphically illustrated in box plots and assessed using *t* test statistics. In addition, Kruskal-Wallis test statistics produced comparable results (*see* Additional file 1: Tables S2 and S3). Because the tests were applied only to the proteins selected with LASSO, *p* values obtained from these analyses were not corrected for multiple testing.

### Statistical Pearson’s correlation and bioinformatic analysis

Correlation analysis was performed on protein fold changes of all 790 quantified proteins using Pearson’s correlation coefficient and Bonferroni correction for multiple comparisons. In addition, Spearman’s correlation analyses produced comparable results (*see* Additional file 1: Tables S4–S6). Several bioinformatics tools and resources were used for analysis and protein annotation (i.e., Database for Annotation, Visualization and Integrated Discovery [DAVID] 6.8 [44], UniProt tissue annotation database [45], Gene Ontology database [46], Kyoto Encyclopedia of Genes and Genomes [KEGG] database [47], tissue atlas [48], and Venny [http://bioinfogp.cnb.csic.es/tools/venny/]).

## Results

### Demographic and clinical characteristics of the study population

Demographics and clinical characteristics of the patient cohort are detailed in Table 1. The cognitively impaired subjects (CDR > 0) were older and less educated and had a higher prevalence of *APOE* ε4 genotype than the cognitively intact group (CDR = 0). In cognitive impairment, CSF Aβ_1–42_ was lower, whereas CSF tau, CSF P-tau181, and CSF P-tau181/Aβ_1–42_ were all higher. MS-based proteomic analyses were performed in the CSF of the 120 individuals (Fig. 1). In total, we measured 790 proteins in CSF. Of those, 541 proteins presented < 5% missing values in 114 subjects (*see* the Methods section above).

The following classification analyses of the CSF P-tau181/Aβ_1–42_ ratios were aimed at separating 39 patients with high-expression AD CSF biomarker profiles (i.e., P-tau181/Aβ_1–42_ > 0.0779) from 75 low-expression profile subjects in the complete analysis set, regardless of the clinical diagnosis. Then, the analyses were performed on the subset of cognitively impaired patients, where 38 and 28 subjects had high and low expression of AD CSF biomarker profiles, respectively.

### Identification of Alzheimer pathology with CSF proteins

**Fig. 2:**
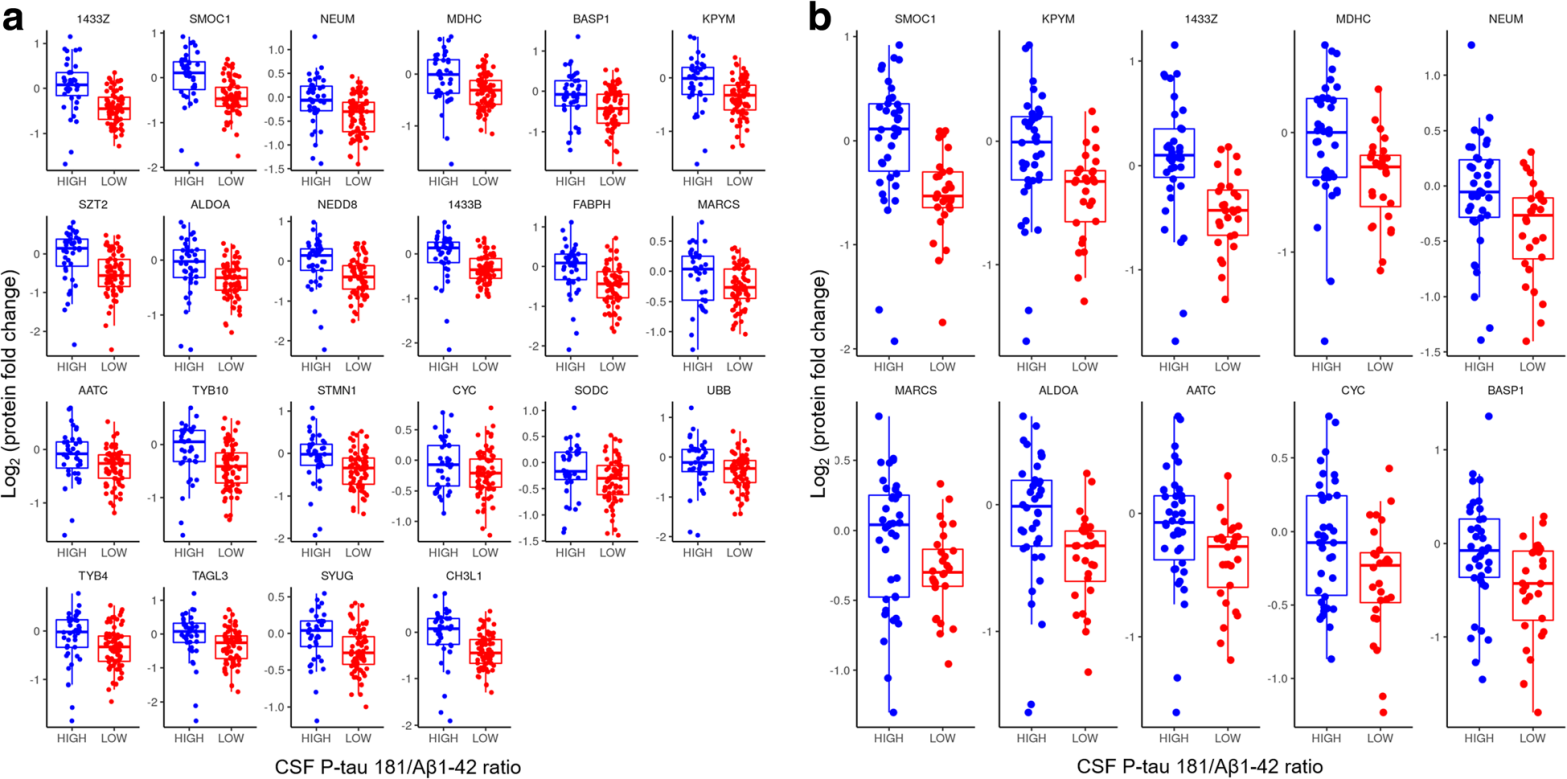
Cerebrospinal fluid (CSF) proteins relevant to Alzheimer pathology. Box plots of CSF proteins according to CSF tau phosphorylated at threonine 181 (P-tau181)/β-amyloid 1–42 (Aβ_1–42_) ratio (i.e., “high” when P-tau181/Aβ_1–42_ > 0.0779 [*blue dots*] and “low” when P-tau181/Aβ_1–42_ ≤ 0.0779 [*red dots*]) for positive and negative CSF profiles of AD pathology, respectively, in all subjects (**a**) and restricted to subjects with cognitive impairment (**b**). In total, 541 CSF proteins were tested (one by one) in a logistic regression model. *P* values were corrected for multiple testing using the Benjamini-Hochberg procedure. Box plots were produced for the significant hits presenting false discovery rate ≤ 5%. Relative protein fold change ratios were used (in Log2). Human proteins in the box plots are given by their UniProtKB/Swiss-Prot entry name

First, we explored whether the CSF proteome presents specific alterations in AD, endophenotypically defined a priori as a CSF P-tau181/Aβ_1–42_ ratio > 0.0779 (*see* the Methods section above). In the whole sample, group comparisons (i.e., “high” when P-tau181/Aβ_1–42_ > 0.0779 and “low” when P-tau181/Aβ_1–42_ ≤ 0.0779) revealed 22 CSF proteins with significant differences between AD versus non-AD CSF biomarker profiles after correction for multiple testing using the Benjamini-Hochberg procedure at FDR ≤ 5% (Fig. 2a and Additional file 1: Table S7). Similarly, in the subset of cognitively impaired subjects (*see* the Methods section above), group comparisons provided ten CSF proteins with significant differences (Fig. 2b and Additional file 1: Table S8). All of these 10 proteins were already present among the 22 proteins (Fig. 2) previously identified in the whole sample.

As a second exploratory approach and ability assessment of the CSF proteome to identify AD, we used LASSO logistic regression to build mathematical models able to classify AD pathology, again defined a priori as a CSF P-tau181/Aβ_1–42_ ratio > 0.0779 (*see* the Methods section above). In the whole sample, the benchmark reference model for classification of CSF P-tau181/Aβ_1–42_ included age and presence of the *APOE* ε4 allele. Its prediction accuracy was 78.3% (as compared with the accuracy of a majority class prediction of 65.8%). CSF protein biomarkers were indeed able to improve the classification of AD CSF biomarker profile with respect to the reference model. The best model accuracy was 100% (McNemar *p* value 3.35 × 10^− 7^). It included 26 CSF proteins (from the 541 provided as input) in addition of age and presence of the *APOE* ε4 allele. Only seven selected CSF proteins displayed significant group comparison differences, i.e., 14-3-3 protein ζ/δ (1433Z) (*p* = 1.69 × 10^− 3^), SPARC-related modular calcium-binding protein 1 (SMOC1) (*p* = 5.26 × 10^− 5^), KICSTOR complex protein SZT2 (SZT2) (*p* = 5.47 × 10^− 4^), fatty acid-binding protein, heart (FABPH) (*p* = 8.70 × 10^− 4^), chitinase-3-like protein 1 (CH3L1) (*p* = 1.23 × 10^− 3^), neuromodulin (NEUM) (*p* = 3.40 × 10^− 3^), and keratin, type I cytoskeletal 10 (*p* = 0.025) (Additional file 1: Figure S1a). Many of these CSF proteins were correlated with each other (Additional file 1: Figure S2). Six of the seven proteins (i.e., 1433Z, SMOC1, SZT2, FABPH, CH3L1, and NEUM) were reported in the exploratory group comparisons (Fig. 2a).

In the subset of cognitively impaired subjects (*see* the Methods section above), the benchmark reference model to classify AD CSF biomarker profile included age, gender, years of education, and presence of *APOE* ε4 allele, with a prediction accuracy of 77.8% (majority class prediction of 57.6%). In cognitive impairment, inclusion of CSF protein biomarkers again improved significantly the prediction accuracy to 100% (McNemar *p* value of 0.0003). In total, 18 CSF proteins (from the 541 provided as input) were included in this best model in addition to gender and presence of the *APOE* ε4 allele. Among those proteins, four displayed significant differences between the groups: 1433Z (*p* = 4.04 × 10^− 5^), SMOC1 (*p* = 5.49 × 10^− 5^), γ-synuclein (*p* = 1.19× 10^− 3^), and macrophage colony-stimulating factor 1 receptor (*p* = 0.013) (Additional file 1: Figure S1b). Again, several correlations were observed between the CSF proteins retained in the model (Additional file 1: Figure S3), suggesting that models with fewer variables may still provide high classification performance. Two of the four proteins (i.e., 1433Z and SMOC1) were reported in the exploratory group comparisons (Fig. 2b). The perfect performance to classify the participants with AD pathology indicated that the reported models were very possibly overfitting the data.

### Associations of CSF proteins with β-amyloid 1–42, tau, and tau phosphorylated at threonine 181

**Fig. 3:**
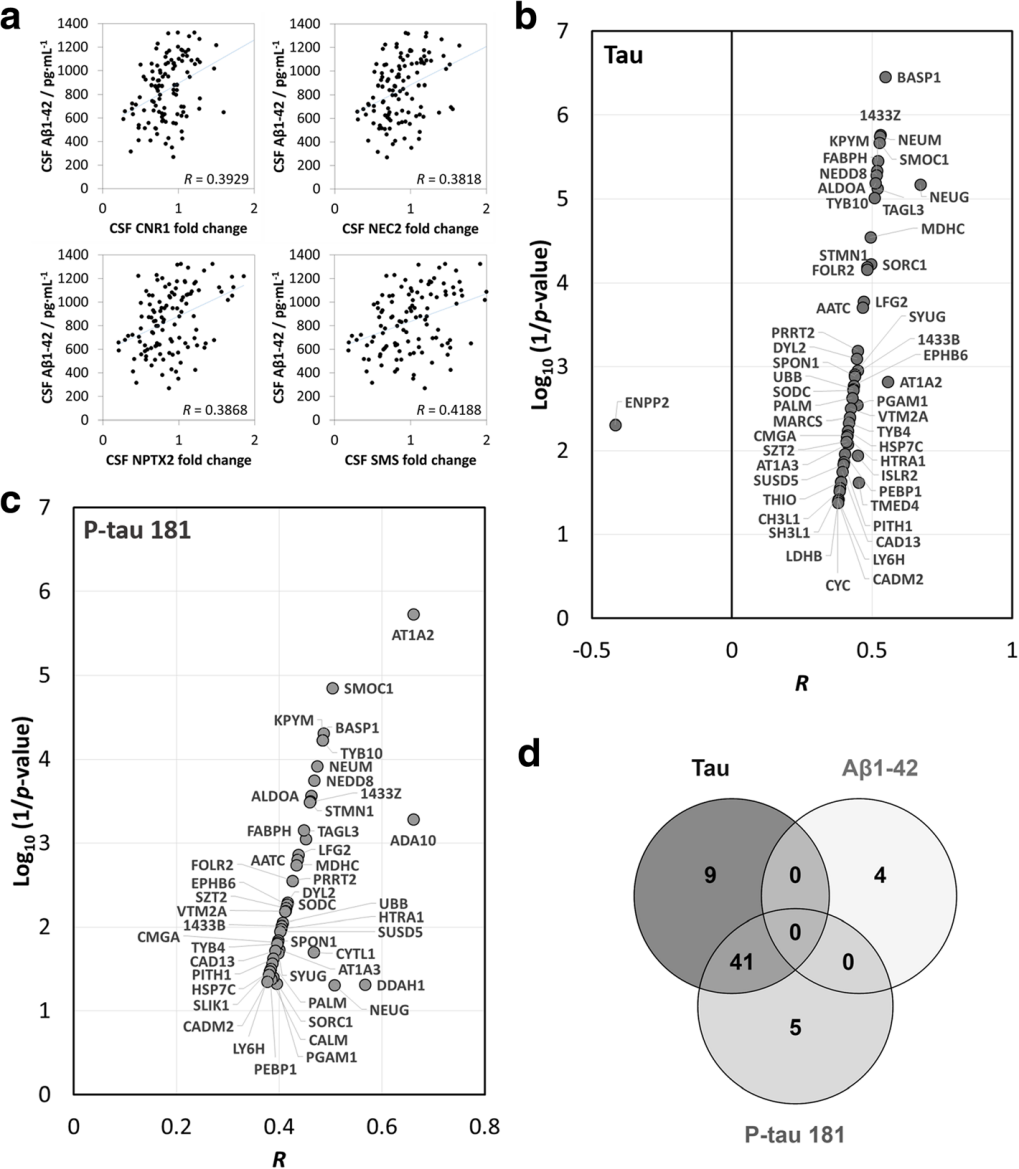
Correlations of cerebrospinal fluid (CSF) proteins with β-amyloid 1–42 (Aβ_1–42_), tau, and tau phosphorylated at threonine 181 (P-tau181) concentrations in CSF. Correlation of CSF proteins with CSF Aβ_1–42_ (**a**), CSF tau (**b**), and CSF P-tau181 (**c**). Only significant correlations with a *p* value ≤ 0.05 after Bonferroni correction for multiple testing were retained and are displayed in the graphs. CSF proteins correlating with CSF Aβ_1–42_, tau, and P-tau181 are illustrated in a Venn diagram (**d**)

Next, we separately and more specifically studied the associations of all 790 quantified CSF proteins (no minimal missing value criteria applied) with CSF markers of core AD pathology (i.e., Aβ_1–42_, tau, and P-tau181). Four proteins—cannabinoid receptor 1 (CNR1, correlation coefficient [*R*] = 0.3929), neuroendocrine convertase 2 (NEC2, *R* = 0.3818), neuronal pentraxin-2 (NPTX2, *R* = 0.3868), and somatostatin (SMS, *R* = 0.4188)—showed an association with CSF Aβ_1–42_, which was significant (*p* value ≤0.05) after Bonferroni correction for multiple testing (Fig. 3a). We found 50 CSF proteins correlated with CSF tau (Fig. 3b) and 46 associated with CSF P-tau181 (Fig. 3c) in a significant manner after Bonferroni correction, of which 41 were in common (Fig. 3d). The five strongest correlations with CSF tau were CSF neurogranin (NEUG), sodium/potassium-transporting ATPase subunit α-2 (AT1A2), brain acid soluble protein 1 (BASP1), 1433Z, and NEUM. The five strongest correlations with CSF P-tau181 were CSF AT1A2, disintegrin and metalloproteinase domain-containing protein 10 (ADA10), *N*^G^,*N*^G^-dimethylarginine dimethylaminohydrolase 1 (DDAH1), NEUG, and SMOC1. In particular, CSF NEUG and NEUM [49], two synaptic proteins, were positively correlated with CSF tau (*R* = 0.6721 and 0.5287, respectively) and P-tau181 (*R* = 0.5074 and 0.4741, respectively) (Additional file 1: Figure S4). All the observed associations are summarized in the chord diagram of Additional file 1: Figure S5. With the exception of ectonucleotide pyrophosphatase/phosphodiesterase family member 2, which negatively correlated with tau, all reported correlations were positive.

### Annotations of CSF proteins correlating with β-amyloid 1–42, tau, and tau phosphorylated at threonine 181

**Fig. 4:**
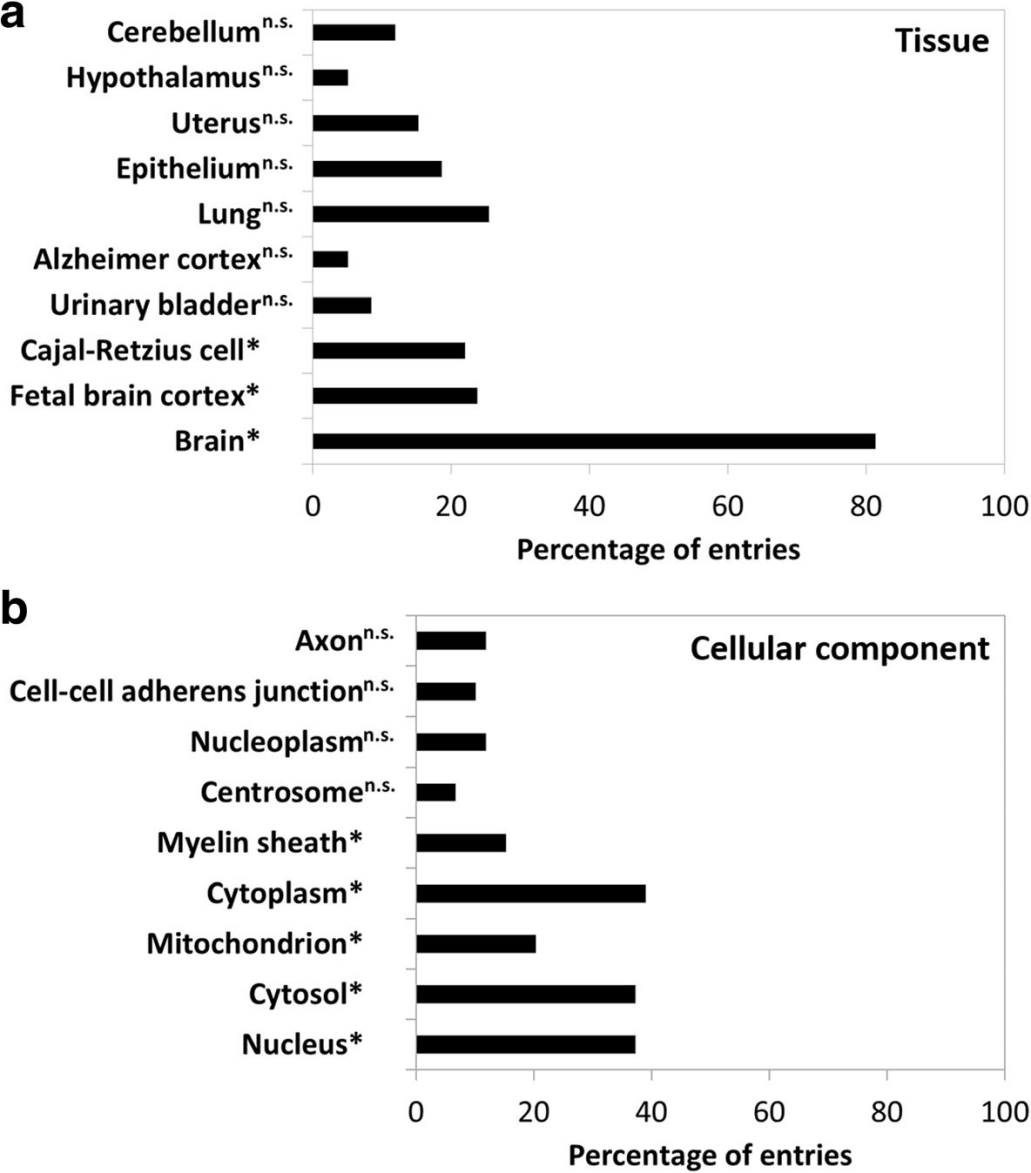
Annotations of cerebrospinal fluid (CSF) proteins correlating with β-amyloid 1–42 (Aβ_1–42_), tau, and/or tau phosphorylated at threonine 181 (P-tau181) concentrations in CSF. Tissue annotation using the UniProt tissue annotation database (**a**) and Gene Ontology (GO) (cellular component category) annotation (**b**) obtained with DAVID software for the 59 CSF proteins correlating with CSF Aβ_1–42_, tau, and/or P-tau181. Significant enrichment (Benjamini-Hochberg procedure) is indicated with an asterisk. The background used for the enrichment analysis was the 790 detected proteins in CSF. *n.s.* Nonsignificant

Of the 59 proteins displaying correlations in those analyses (Fig. 3d), most are expressed in the brain, in particular in the fetal brain cortex and Cajal-Retzius cells (Fig. 4a). Moreover, and based on the tissue-based map of the human proteome [48], seven proteins (i.e., SLIT and NTRK-like protein 1, NEUM, NEUG, cell adhesion molecule 2, lymphocyte antigen 6H [LY6H], transgelin-3 [TAGL3], and protein lifeguard) are brain-enriched (i.e., having at least fivefold higher mRNA levels in the brain as compared with all other tissues) and a total of 22 proteins have elevated gene expression in the brain (i.e., in addition to the seven above, AT1A2, immunoglobulin superfamily containing leucine-rich repeat protein 2 [ISLR2], sodium/potassium-transporting ATPase subunit α-3 [AT1A3], BASP1, CH3L1, CNR1, ephrin type-B receptor 6 [EPHB6], NPTX2, paralemmin-1, NEC2, proline-rich transmembrane protein 2, SMOC1, VPS10 domain-containing receptor SorCS1, SMS, and V-set and transmembrane domain-containing protein 2A).

In Fig. 4b, we identified the myelin sheath as an enriched cellular component. Of the 59 CSF proteins correlating with Aβ_1–42_, tau, and/or P-tau181, 9 proteins pertain to the myelin sheath: TAGL3, malate dehydrogenase, cytoplasmic (MDHC), heat shock cognate 71 kDa protein (HSP7C), AT1A2, phosphoglycerate mutase 1 (PGAM1), superoxide dismutase [Cu-Zn] (SODC), AT1A3, pyruvate kinase PKM (KPYM), and L-lactate dehydrogenase B chain (LDHB). Those nine proteins were associated with tau and/or P-tau181. Pathway enrichment analysis using the KEGG database did not yield any significant results (data not shown).

## Discussion

In the present study, we used MS-based shotgun proteomics to measure the CSF proteomes of 120 older adults and investigate broad CSF protein relationships with core AD pathology. Overall, human CSF proteome coverage was composed of 790 proteins. Four CSF proteins were associated with CSF Aβ_1–42_ levels, 50 proteins with CSF tau, and 46 proteins with CSF P-tau181 levels. The CSF proteins related to Aβ_1–42_ were different from those associated with tau or P-tau181.

To explore the relevance of the CSF proteome to AD pathology, we applied an approach that was unbiased by the clinical diagnosis and defined endophenotypically the disease as the presence of “core” AD pathology (i.e., the combined presence of cerebral amyloid and tau pathology). Unbiased classification based on markers of cerebral amyloid and tau pathology and neuronal injury has been proposed for use across the clinical stages [7]. We first used two exploratory approaches to evaluate and select CSF proteins that were able to stratify subjects according to levels of CSF P-tau181/Aβ_1–42_. Using LASSO logistic regression, we observed that CSF proteins could significantly increase the classification accuracy of non-AD versus AD CSF biomarker profiles as compared with models based only on clinical parameters and the presence of the *APOE* ε4 allele. Nonetheless, those statistical models relying on CSF proteins might be overfitted and should be interpreted with caution; class imbalance also affected their strict performance. Overall, with both exploratory analyses, we identified specific CSF proteome alterations that are related to AD pathology and may provide novel mechanistic insights. Assessing the whole sample and the subgroup of subjects with cognitive impairment, we could decipher the strong contribution of some CSF proteins, such as SMOC1 and 1433Z (Fig. 2 and Additional file 1: Figure S1). On the basis of this performance, we specifically investigated associations of CSF proteins with individual most validated biomarkers of amyloid pathology, neuronal injury, and tau hyperphosphorylation (i.e., Aβ_1–42_, tau, and P-tau181, respectively) to elaborate further on the involved mechanisms. Most of the correlations of CSF proteins were with CSF tau and P-tau181 (Fig. 3d), suggesting the CSF proteome alterations to be more representative of tau pathology than amyloid pathology. Four CSF proteins not related to tau and P-tau181 were associated with CSF Aβ_1–42_ levels, overall indicating distinct proteome alterations related to either amyloid pathology or tau-related neurodegeneration. The majority of these proteins were brain-enriched proteins, including synaptic proteins, and proteins involved in reelin-producing cells and the myelin sheath. Comparison of the proteins found with different levels in AD versus non-AD CSF biomarker profiles and in the models able to classify CSF-defined AD pathology with those associated with CSF Aβ_1–42_, tau, and P-tau181 in Venn diagrams (Additional file 1: Figures S6 and S7, respectively) revealed mixed overlaps. Interestingly, the 22 proteins with different levels in AD versus non-AD CSF biomarker profiles (Fig. 2a) were all associated with CSF tau; a large majority were associated with CSF P-tau181; but none were associated with CSF Aβ_1–42_ (Additional file 1: Figure S6). Nevertheless, beyond those 22 proteins, 37 proteins, still representing the majority of CSF proteins associated with CSF Aβ_1–42_, tau, and P-tau181, were not evidenced as having a relationship to AD, suggesting they might represent more general makers of amyloid pathology, neuronal injury, and tau hyperphosphorylation.

The CSF proteins CNR1, NEC2, NPTX2, and SMS were associated with CSF Aβ_1–42_ in our study (Fig. 3a). CNR1 and the endocannabinoid system were previously identified as potential targets for treatment of neurological disorders and AD in particular [50, 51]. In line with our results, higher NPTX2, a proinflammatory protein involved in synaptic plasticity, was previously associated with higher CSF Aβ_1–42_ in the Alzheimer’s Disease Neuroimaging Initiative study [52]. NEC2, also known as prohormone convertase 2, is essential to the processing of pro-islet amyloid polypeptide [53]. Its role in the processing of hormones and in particular of neuropeptide precursors in the human cortex has been established, but the link with SMS deficiency in AD, for instance, was not confirmed [54]. Relevant to our observations, neuropeptide SMS is known to be decreased in the CSF of patients with AD [55] and to regulate Aβ_1–42_ via proteolytic degradation [56]. Together, these findings indicate amyloid-related changes in the CSF proteome that may be particularly relevant for early cerebral AD pathology as well as for disease-modifying interventions targeting amyloid and starting at preclinical disease stages.

We found that CSF Aβ_1–42_, tau, and P-tau181 were mainly associated with CSF proteins enriched in brain tissue (Fig. 4a), and this despite the important proportion (about 80%) of proteins in CSF originating from blood [4]. In particular, some are expressed in the fetal brain cortex. We observed positive correlations between CSF tau and/or P-tau181 with 13 CSF proteins (i.e., calmodulin, fructose-bisphosphate aldolase A [ALDOA], DDAH1, HSP7C, KPYM, LDHB, MDHC, PGAM1, phosphatidylethanolamine-binding protein 1 [PEBP1], stathmin, TAGL3, thioredoxin, and 1433Z) known also to be present in reelin-producing Cajal-Retzius cells. In early AD, a massive decline of the number of Cajal-Retzius cells was previously described [57], suggesting a link between their loss, reduction of reelin, impairment of synaptic plasticity, amyloid plaque deposition, and neurofibrillary tangle formation [58]. Interestingly, we also revealed the involvement of nine CSF proteins (i.e., AT1A2, AT1A3, HSP7C, KPYM, LDHB, MDHC, PGAM1, SODC, and TAGL3), again positively correlating with CSF tau and/or P-tau181, being specifically part of the myelin sheath. Although amyloid plaques and neurofibrillary tangles likely induce neuronal and synaptic loss, myelin alteration may also participate in the development of AD dementia. Myelin content changes in the white matter measured with MRI have been linked to CSF AD biomarkers (i.e., lower concentrations of Aβ_1–42_ and higher concentrations of tau and P-tau181), but mainly in association with amyloid pathology [59]. Our results, including associations of AT1A2 and KPYM with both tau and P-tau181, may suggest an underestimated connection between tau-related neurodegeneration and (de)myelination. These specific alterations provide new insights into the disease pathology and deserve further exploration.

Several single relationships between CSF proteins and Aβ_1–42_, tau, and/or P-tau181 levels in our study (Fig. 3) have previously been reported. A first example is the synaptic protein NEUG, which was previously proposed as a novel candidate CSF biomarker for AD and prodromal AD; high CSF NEUG was shown to predict future cognitive decline and to be more specific for AD than tau [60]. In addition, CSF NEUG was reported to be increased in AD and positively correlated with CSF tau [61] and P-tau [49]. In line with our observations, positive associations were identified with NEUM for both tau and P-tau in CSF [49]. BASP1, like NEUM, is a presynaptic membrane protein participating in axon guidance, neurodegeneration, and synaptic plasticity [62] and was found to be significantly downregulated in AD versus control brain samples [63]. Our findings of significant association of CSF BASP1 with both CSF tau and P-tau warrant further investigations. Mutations in the *ADAM10* gene, which encodes the major α-secretase responsible for cleaving APP, have previously been identified in families with late-onset AD [64]. In our study, protein ADA10, which is encoded by *ADAM10*, was only significantly associated with CSF P-tau181. To the best of our knowledge, such an association between those CSF proteins has not been observed before [65].

Further and broader cross-validation of our findings can be made by comparing them with those of a recent study investigating CSF proteins associated with CSF AD biomarkers in 58 cognitively healthy men using an aptamer-based technology (i.e., SOMAscan; SomaLogic, Boulder, CO, USA) [66]. Of the 59 CSF proteins associated with CSF biomarkers of core AD pathology that we report, 28 were also measured with the SOMAscan in that prior study; of those, 22 proteins (i.e., 78.6% overlap) were correlated with CSF Aβ_1–42_, tau, and/or P-tau [66], confirming part of our observations in an independent cohort and using a different technology. Those proteins are ALDOA, dynein light chain 2, cytoplasmic, polyubiquitin B, ISLR2, EPHB6, MDHC, SH3 domain-binding glutamic acid-rich-like protein, PEBP1, NPTX2, chromogranin A, cytochrome c, SMS, 1433Z, LDHB, SMOC1, 14–3-3 protein β/α, spondin-1, FABPH, transmembrane emp24 domain-containing protein 4, PGAM1, cytokine-like protein 1, and HSP7C.

Altogether, our shotgun MS-based proteomic approach [22] was confirmed to provide relevant findings and to be complementary to alternative proteomic technologies. In this perspective, the identification of novel and strongly significant associations of CSF proteins with CSF biomarkers of AD core pathology in our study is of specific interest. In particular, proteins AT1A2 and KPYM implicated in energy production, as well as 1433Z, DDAH1, and SMOC1, showing some of the strongest associations with tau and/or P-tau181 in addition to NEUG and NEUM, could appear relevant. Our results in a relatively large group of subjects including both participants with cognitive impairment and healthy volunteers are therefore encouraging. Sample fractionation would have allowed deeper proteome coverage but with a throughput incompatible with the analysis of 120 clinical samples in a reasonable time frame. The proteins we have identified would deserve additional research.

## Conclusions

Using an MS-based proteomic workflow, we have quantified a number of CSF proteins in 120 older adults with normal cognition and with cognitive impairment. We report strong evidence of known and new CSF proteins related to amyloid pathology, neuronal injury, and tau hyperphosphorylation. Although we confirmed several previous findings of CSF proteins related to AD pathology, our work reveals a large number of additional CSF proteome alterations involving in particular reelin-producing cells and the myelin sheath.

## Additional file

Additional file 1: Supplementary Methods. **Table S1.** Demographics and clinical characteristics of subjects removed from the statistical analyses. **Table S2.** Non-AD versus AD CSF biomarker profile group comparison after selection in all subjects of 26 proteins with LASSO. **Table S3.** Non-AD versus AD CSF biomarker profile group comparison after selection in subjects with cognitive impairment of 18 proteins with LASSO. **Table S4.** Correlation of CSF proteins with CSF Aβ1-42. **Table S5.** Correlation of CSF proteins with CSF tau. **Table S6.** Correlation of CSF proteins with CSF P-tau181. **Table S7.** Group comparisons of CSF protein measurements for AD versus non-AD CSF biomarker profiles in all subjects. **Table S8.** Group comparisons of CSF protein measurements for AD versus non-AD CSF biomarker profiles in subjects with cognitive impairment. **Figure S1.** Box-plots of CSF proteins (selected with LASSO analyses) for positive and negative CSF profiles of AD pathology in all subjects and subjects with cognitive impairment. **Figure S2.** Pairwise correlation heatmap of the 26 CSF proteins selected with LASSO for classification of non-AD versus AD CSF biomarker profiles for all subjects. **Figure S3.** Pairwise correlation heatmap of the 18 CSF proteins selected with LASSO for classification of non-AD versus AD CSF biomarker profiles for subjects with cognitive impairment. **Figure S4.** Correlations of CSF neurogranin and neuromodulin with CSF tau and P-tau181. **Figure S5.** Chord diagram of the relationships of 59 CSF proteins with CSF tau, P-tau181, and/or Aβ1-42. **Figure S6.** Venn diagrams of CSF proteins with significant group comparison differences between AD versus non-AD CSF biomarker profiles and those correlating with CSF Aβ1-42, tau, and P-tau181. **Figure S7.** Venn diagrams of CSF proteins selected with LASSO to classify non-AD versus AD CSF biomarker profiles and those correlating with CSF Aβ1-42, tau, and P-tau181. (DOCX 2575 kb)

## Abbreviations

1433B: 14-3-3 protein β/α
2LFG2: Protein lifeguard
AD: Alzheimer disease
ADA10: Disintegrin and metalloproteinase domain-containing protein 10
ALDOA: Fructose-bisphosphate aldolase A
*APOE*: Apolipoprotein E
AT1A2: Sodium/potassium-transporting ATPase subunit α-2
AT1A3: Sodium/potassium-transporting ATPase subunit α-3
AUC: Area under the curve
BASP1: Brain acid soluble protein 1
CADM2: Cell adhesion molecule 2
CALM: Calmodulin
CDR: Clinical Dementia Rating
CH3L1: Chitinase-3-like protein 1
CMGA: Chromogranin-A
CNR1: Cannabinoid receptor 1
CNS: Central nervous system
CSF: Cerebrospinal fluid
DDAH1: *N*^G^,*N*^G^-dimethylarginine dimethylaminohydrolase 1
CSF1R: Macrophage colony-stimulating factor 1 receptor
CYC: Cytochrome c
CYTL1: Cytokine-like protein 1
DYL2: Dynein light chain 2, cytoplasmic
ENPP2: Ectonucleotide pyrophosphatase/phosphodiesterase family member 2
EPHB6: Ephrin type- B receptor 6
FABPH: Fatty acid-binding protein, heart
FDR: False discovery rate
HSP7C: Heat shock cognate 71 kDa protein
ISLR2: Immunoglobulin superfamily containing leucine-rich repeat protein 2
K1C10: Keratin, type I cytoskeletal 10
KPYM: Pyruvate kinase PKM
LASSO: Least absolute shrinkage and selection operator regression
LC: Liquid chromatography
LDHB: L-lactate dehydrogenase B chain
MCI: Mild cognitive impairment
MDHC: Malate dehydrogenase, cytoplasmic
MMSE: Mini Mental State Examination
MS: Mass spectrometry
MS/MS: Tandem mass spectrometry
MRI: Magnetic resonance imaging
NEC2: Neuroendocrine convertase 2
NEUG: Neurogranin
NEUM: Neuromodulin
NPTX2: Neuronal pentraxin-2
PALM: Paralemmin-1
PEBP1: Phosphatidylethanolamine-binding protein 1
PGAM1: Phosphoglycerate mutase 1
PRRT2: Proline-rich transmembrane protein 2
P-tau: Hyperphosphorylated tau
P-tau181: Tau phosphorylated at threonine 181
RP: Reversed-phase
SLIK1: SLIT and NTRK-like protein 1
SMOC1: SPARC-related modular calcium-binding protein 1
SMS: Somatostatin
SODC: Superoxide dismutase [Cu-Zn]
SORC1: VPS10 domain-containing receptor SorCS1
SPON1: Spondin-1
STMN1: Stathmin
SYUG: γ-Synuclein
SZT2: KICSTOR complex protein SZT2
TAGL3: Lymphocyte antigen 6H (LY6H), transgelin-3
Aβ_1–-42_: β-Amyloid 1–-42
1433Z: 14-3-3 protein ζ/δ
