# Supplementary material for: Alzheimer disease pathology and the cerebrospinal fluid proteome
Source: Alzheimers Res Ther. 2018 Jul 18;10:66. doi: 10.1186/s13195-018-0397-4 (PMC6052524; doi:10.1186/s13195-018-0397-4)
Supplement: Supplementary file 1 — Table S1. Demographics and clinical characteristics of subjects removed from the statistical analyses. Table S2. Non-AD versus AD CSF biomarker profile group comparison after selection in all subjects of 26 proteins with LASSO. Table S3. Non-AD versus AD CSF biomarker profile group comparison after selection in subjects with cognitive impairment of 18 proteins with LASSO. Table S4. Correlation of CSF proteins with CSF Aβ1-42. Table S5. Correlation of CSF proteins with CSF tau. Table S6. Correlation of CSF proteins with CSF P-tau181. Table S7. Group comparisons of CSF protein measurements for AD versus non-AD CSF biomarker profiles in all subjects. Table S8. Group comparisons of CSF protein measurements for AD versus non-AD CSF biomarker profiles in subjects with cognitive impairment. Figure S1. Box-plots of CSF proteins (selected with LASSO analyses) for positive and negative CSF profiles of AD pathology in all subjects and subjects with cognitive impairment. Figure S2. Pairwise correlation heatmap of the 26 CSF proteins selected with LASSO for classification of non-AD versus AD CSF biomarker profiles for all subjects. Figure S3. Pairwise correlation heatmap of the 18 CSF proteins selected with LASSO for classification of non-AD versus AD CSF biomarker profiles for subjects with cognitive impairment. Figure S4. Correlations of CSF neurogranin and neuromodulin with CSF tau and P-tau181. Figure S5. Chord diagram of the relationships of 59 CSF proteins with CSF tau, P-tau181, and/or Aβ1-42. Figure S6. Venn diagrams of CSF proteins with significant group comparison differences between AD versus non-AD CSF biomarker profiles and those correlating with CSF Aβ1-42, tau, and P-tau181. Figure S7. Venn diagrams of CSF proteins selected with LASSO to classify non-AD versus AD CSF biomarker profiles and those correlating with CSF Aβ1-42, tau, and P-tau181. (DOCX 2575 kb) [file 13195_2018_397_MOESM1_ESM.docx]

**ADDITIONAL FILE**

**Alzheimer disease pathology and the cerebrospinal fluid proteome**

Loïc Dayon^1, *^, Antonio Núñez Galindo^1^, Jérôme Wojcik^2^, Ornella Cominetti^1^, John Corthésy^1^, Aikaterini Oikonomidi^3^, Hugues Henry^4^, Martin Kussmann^1, $^, Eugenia Migliavacca^1^, India Severin^1^, Gene L. Bowman^1^, and Julius Popp^3^

^1^Nestlé Institute of Health Sciences, Lausanne, Switzerland

^2^Precision for Medicine, Geneva, Switzerland

^3^CHUV, Old Age Psychiatry, Department of Psychiatry, Lausanne, Switzerland

^4^CHUV, Department of Laboratories, Lausanne, Switzerland

^$^Current affiliation: Liggins Institute, University of Auckland, New Zealand

**Email:** [loic.dayon@rd.nestle.com](mailto:loic.dayon@rd.nestle.com); [antonio.nunezgalindo@rd.nestle.com](mailto:antonio.nunezgalindo@rd.nestle.com); [jerome.wojcik@precisionformedicine.com](mailto:jerome.wojcik@precisionformedicine.com); [ornella.cominetti@rd.nestle.com](mailto:ornella.cominetti@rd.nestle.com); [john.corthesy@rd.nestle.com](mailto:john.corthesy@rd.nestle.com); [oikonomidi@yahoo.com](mailto:oikonomidi@yahoo.com); [hugues.henry@chuv.ch](mailto:hugues.henry@chuv.ch); [m.kussmann@auckland.ac.nz](mailto:m.kussmann@auckland.ac.nz); [eugenia.migliavacca@rd.nestle.com](mailto:eugenia.migliavacca@rd.nestle.com); [india.severin@rd.nestle.com](mailto:india.severin@rd.nestle.com); [gene.bowman@rd.nestle.com](mailto:gene.bowman@rd.nestle.com); [julius.popp@chuv.ch](mailto:julius.popp@chuv.ch)

**Correspondence to:** *Nestlé Institute of Health Sciences, EPFL Innovation Park, Bâtiment H, 1015 Lausanne, Switzerland; Email: [loic.dayon@rd.nestle.com](mailto:loic.dayon@rd.nestle.com), Phone: +41 21 632 6114, Fax: +41 21 632 6499

**SUPPLEMENTARY METHODS**

***Materials for proteomic analysis***

Iodoacetamide (IAA), tris(2-carboxyethyl) phosphine hydrochloride (TCEP), triethylammonium hydrogen carbonate buffer 1 M pH = 8.5, sodium dodecyl sulfate, and β-lactoglobulin (LACB) from bovine milk were purchased from Sigma (St. Louis, MO, USA). Formic acid (FA, 99%) and CH_3_CN were from BDH (VWR International Ltd., Poole, UK). Hydroxylamine solution 50 *wt* % in H_2_O (99.999%) was acquired from Aldrich (Milwaukee, WI, USA). H_2_O (18.2 MΩ·cm at 25 °C) was obtained from a Milli-Q apparatus (Millipore, Billerica, MA, USA). Trifluoroacetic acid Uvasol^®^ was sourced from Merck Millipore (Billerica, MA, USA). The 6-plex tandem mass tags (TMTs) [[37](#_ENREF_37)] were purchased from Thermo Scientific (Rockford, IL, USA). Sequencing grade modified Lys-C/trypsin was procured from Promega (Madison, WI, USA). For immuno-affinity depletion of 14 abundant human proteins, multiple affinity removal system (MARS) columns, Buffer A, and Buffer B were obtained from Agilent Technologies (Wilmington, DE, USA). Oasis HLB cartridges (1cc, 30 mg) were acquired from Waters (Milford, MA, USA) and Strata-X 33u Polymeric reversed-phase (RP) and Strata-X-C 33u Polymeric strong cation-exchange (SCX) solid-phase extraction (SPE) cartridges (30 mg/1 mL) from Phenomenex (Torrance, CA, USA).

***Sample preparation for mass spectrometry-based proteomics***

CSF samples were prepared as previously described [[36](#_ENREF_36)]. A volume of 400 µL (for 12 samples, this volume was not available; different volumes were therefore taken and correction factors were subsequently applied) of CSF sample was evaporated with a *vacuum* centrifuge. The dried CSF samples were diluted in 125 µL of depletion Buffer A containing 0.00965 mg·mL^−1^ LACB. All samples were filtered using a 0.22 µm filter plate (Millipore).

Immuno-affinity depletion was performed by removing 14 highly abundant proteins from the 100 µL filtered CSF sample solutions. Samples were depleted with MARS columns, following the manufacturer instructions and using high performance liquid chromatography (LC) systems (Thermo Scientific, San Jose, CA, USA) equipped with HTC-PAL (CTC Analytics, Zwingen, Switzerland) fraction collectors. After immuno-depletion, samples were snap-frozen and stored at -80 °C. Buffer exchange was performed with RP cartridges mounted on a 96-hole holder and a *vacuum* manifold as previously described [[38](#_ENREF_38)]. Samples were subsequently evaporated with a *vacuum* centrifuge (Thermo Scientific) and stored at -80 °C. Reduction with TCEP, alkylation with IAA, digestion with Lys-C/trypsin, TMT 6-plex labeling, sample pooling, and SPE purification (Oasis HLB and SCX) were performed on a 4-channels Microlab Star liquid handler (Hamilton, Bonaduz, Switzerland) according to a previously reported protocol [[38](#_ENREF_38)]. The pooled 6-plex TMT-labeled samples were then evaporated to dryness before storage at -80 °C.

***Proteomic analysis with reversed-phase liquid chromatography mass spectrometry***

The CSF samples were dissolved in 200 µL H_2_O/CH_3_CN/FA 96.9/3/0.1, for RP-LC tandem mass spectrometry (MS/MS). RP-LC MS/MS was performed with a hybrid linear ion trap-Orbitrap (OT) Elite and an Ultimate 3000 RSLC nano system (Thermo Scientific) as recently described [[38](#_ENREF_38)]. Proteolytic peptides (injection of 5 µL of sample) were trapped on an Acclaim PepMap 75 µm × 2 cm (C18, 3 µm, 100 Å) pre-column and separated on an Acclaim PepMap RSLC 75 µm × 50 cm (C18, 2 µm, 100 Å) column (Thermo Scientific) coupled to a stainless steel nanobore emitter (40 mm, OD 1/32”) mounted on a Nanospray Flex Ion Source (Thermo Scientific). The analytical separation was run for 150 min using a gradient that reached 30% of CH_3_CN after 140 min and 80% of CH_3_CN after 150 min at a flow rate of 220 nL·min^−1^ (total analysis time was 180 min). For MS survey scans, the OT resolution was 120000 (ion population of 1 × 10^6^) with an *m/z* window from 300 to 1500. For MS/MS with higher-energy collisional dissociation at 35% of the normalized collision energy, ion population was set to 1 × 10^5^ (isolation width of 2), with a resolution of 15000, first mass at *m/z* = 100, and a maximum injection time of 250 ms in the OT. A maximum of 10 precursor ions (most intense) were selected for MS/MS. Dynamic exclusion was set for 60 s within a ± 5 ppm window. A lock mass of *m/z* = 445.1200 was used. Each sample was analyzed in duplicate.

***Mass spectrometry-based proteomic data analysis***

Proteome Discoverer (version 1.4, Thermo Scientific) was used as data analysis interface. Identification was performed against the human UniProtKB/Swiss-Prot database (08/12/2014 release) including the LACB sequence (20194 sequences in total). Mascot (version 2.4.2, Matrix Sciences, London, UK) was used. Variable amino acid modifications were oxidized methionine, deamidated asparagine/glutamine, and 6-plex TMT-labeled peptide amino terminus. 6-plex TMT-labeled lysine was set as fixed modifications as well as carbamidomethylation of cysteine. Trypsin was selected as the proteolytic enzyme, with a maximum of two potential missed cleavages. Peptide and fragment ion tolerance were set to, respectively, 10 ppm and 0.02 Da. All Mascot result files were loaded into Scaffold Q+S 4.4.1.1 (Proteome Software, Portland, OR, USA) to be further searched with X! Tandem (version CYCLONE (2010.12.01.1), the GPM, <http://thegpm.org/>). Both peptide and protein false discovery rates were fixed at 1% maximum, with a two unique peptide criterion to report protein identification. Quantitative values were exported from Scaffold Q+S as log_2_ of the ratio fold changes with respect to their measurements in the biological reference (**Fig. 1**), *i.e.*, mean log_2_ values after isotopic purity correction but without normalization applied between samples and experiments. The biological reference was a pool of all individual CSF samples labelled with 6-plex TMT reporter-ions at *m/z* = 126 and 131, allowing fold change calculation with respect to both channels. After confirming a good agreement between the calculation results, data from both replicates and references were averaged to generate one single data matrix.

***CSF β-amyloid 1-42, tau, tau phosphorylated at threonine 181, and APOE genotyping***

CSF Aβ1-42, tau, and P-tau 181 concentrations were measured using commercially available enzyme-linked immunosorbent assay kits (Fujirebio, Ghent, Belgium).

Deoxyribonucleic acid (DNA) was extracted from whole blood using the QIAsymphony DSP DNA Kit (Qiagen, Hombrechtikon, Switzerland) as previously described [[69](#_ENREF_69)]. The single nucleotide variant rs429358 and rs7412 were genotyped using the Taqman assays C___3084793_20 and C____904973_10, respectively (Thermo Fischer Scientific, Waltham, MA, USA).

**REFERENCES**

36. Núñez Galindo A, Kussmann M, Dayon L. Proteomics of Cerebrospinal Fluid: Throughput and Robustness Using a Scalable Automated Analysis Pipeline for Biomarker Discovery. Anal Chem. 2015;87:10755-61.

37. Dayon L, Sanchez JC: Relative protein quantification by MS/MS using the tandem mass tag technology. In *Methods in Molecular Biology*, vol. 893. pp. 115-27; 2012:115-27.

38. Dayon L, Núñez Galindo A, Corthésy J, Cominetti O, Kussmann M. Comprehensive and scalable highly automated MS-based proteomic workflow for clinical biomarker discovery in human plasma. J Proteome Res. 2014;13:3837-45.

67. Dayon L, Wojcik J, Núñez Galindo A, Corthésy J, Cominetti O, Oikonomidi A, et al. Plasma proteomic profiles of cerebrospinal fluid-defined Alzheimer’s disease pathology in older adults. J Alzheimers Dis. 2017;60:1641-52.

**Table S1.** Demographics and clinical characteristics of the six subjects remove from the statistical analyses because of their CSF samples presenting aberrant proteomic values.

| **Gender** | **Age (years)** | **CDR** | **MMSE** | **CSF profile of AD pathology** |
| --- | --- | --- | --- | --- |
| Female | 76 | 0.5 | 25 | yes |
| Male | 76 | 0.5 | 27 | no |
| Male | 84 | 0.5 | 28 | yes |
| Female | 80 | 0.5 | 22 | no |
| Female | 79 | 0.5 | 20 | no |
| Female | 75 | 0.5 | 29 | yes |

**Table S2.** Non-AD *versus* AD CSF biomarker profile group comparison after selection in all subjects of 26 proteins with **LASSO** analysis. *P*-values and ranks are presented.

| **MARKER** | **TTEST_P** | **TTEST_RANK** | **KRUSKAL_P** | **KRUSKAL_RANK** |
| --- | --- | --- | --- | --- |
| 1433Z_HUMAN | 1.688e-05 | 1 | 3.226e-07 | 1 |
| SMOC1_HUMAN | 5.255e-05 | 2 | 5.163e-07 | 2 |
| CH3L1_HUMAN | 0.001234 | 5 | 4.065e-06 | 3 |
| FABPH_HUMAN | 0.0008697 | 4 | 2.647e-05 | 4 |
| SZT2_HUMAN | 0.000547 | 3 | 2.79e-05 | 5 |
| NEUM_HUMAN | 0.003398 | 6 | 0.000907 | 6 |
| K1C10_HUMAN | 0.02524 | 7 | 0.04508 | 7 |
| TMG1_HUMAN | 0.09502 | 8 | 0.05333 | 8 |
| CHAD_HUMAN | 0.1891 | 10 | 0.434 | 16 |
| HBB_HUMAN | 0.3138 | 13 | 0.4218 | 15 |
| GLDN_HUMAN | 0.7121 | 20 | 0.09623 | 9 |
| CBPE_HUMAN | 0.5656 | 16 | 0.3848 | 14 |
| WFDC1_HUMAN | 0.2773 | 12 | 0.468 | 18 |
| GLT10_HUMAN | 0.7169 | 21 | 0.2381 | 10 |
| PLTP_HUMAN | 0.5353 | 15 | 0.468 | 17 |
| SODE_HUMAN | 0.6848 | 19 | 0.3592 | 13 |
| MYG_HUMAN | 0.1497 | 9 | 0.8461 | 24 |
| SFRP4_HUMAN | 0.2004 | 11 | 0.6956 | 23 |
| LRP1_HUMAN | 0.8716 | 25 | 0.2918 | 11 |
| RELN_HUMAN | 0.3451 | 14 | 0.665 | 22 |
| LPHN3_HUMAN | 0.8962 | 26 | 0.3438 | 12 |
| MOG_HUMAN | 0.7902 | 23 | 0.4791 | 19 |
| WFKN2_HUMAN | 0.5988 | 17 | 0.8461 | 25 |
| HRG_HUMAN | 0.7709 | 22 | 0.6477 | 21 |
| CUTA_HUMAN | 0.8378 | 24 | 0.5131 | 20 |
| NPTX2_HUMAN | 0.6153 | 18 | 0.9643 | 26 |

**Table S3.** Non-AD *versus* AD CSF biomarker profile group comparison after selection in subjects with cognitive impairment of 18 proteins with **LASSO** analysis. *P*-values and ranks are presented.

| **MARKER** | **TTEST_P** | **TTEST_RANK** | **KRUSKAL_P** | **KRUSKAL_RANK** |
| --- | --- | --- | --- | --- |
| SMOC1_HUMAN | 5.491e-05 | 2 | 1.091e-05 | 1 |
| 1433Z_HUMAN | 4.043e-05 | 1 | 1.751e-05 | 2 |
| SYUG_HUMAN | 0.001195 | 3 | 0.0008549 | 3 |
| CSF1R_HUMAN | 0.01321 | 4 | 0.02318 | 4 |
| ATS8_HUMAN | 0.05275 | 5 | 0.02564 | 5 |
| HELZ_HUMAN | 0.1063 | 6 | 0.2177 | 7 |
| DHPR_HUMAN | 0.4922 | 10 | 0.1426 | 6 |
| K1C10_HUMAN | 0.1318 | 7 | 0.2815 | 11 |
| CNDP1_HUMAN | 0.6084 | 13 | 0.2226 | 8 |
| SFRP4_HUMAN | 0.1953 | 8 | 0.6312 | 15 |
| KLK6_HUMAN | 0.6611 | 14 | 0.2482 | 10 |
| NPTX2_HUMAN | 0.6042 | 12 | 0.4212 | 13 |
| WFKN2_HUMAN | 0.824 | 16 | 0.2226 | 9 |
| FIBA_HUMAN | 0.3501 | 9 | 0.9586 | 18 |
| WFDC1_HUMAN | 0.5474 | 11 | 0.907 | 16 |
| LPHN3_HUMAN | 0.8282 | 17 | 0.3707 | 12 |
| AUGN_HUMAN | 0.7497 | 15 | 0.9586 | 17 |
| MOG_HUMAN | 0.9863 | 18 | 0.5165 | 14 |

**Table S4.** Correlation of CSF proteins with CSF Aβ1-42 (significant Pearson hits). Level of significance is indicated as *p*-value after Bonferroni correction.

| **BIOMARKER** | **PEARSON_P** | **PEARSON_RANK** | **SPEARMAN_P** | **SPEARMAN_RANK** |
| --- | --- | --- | --- | --- |
| SMS_HUMAN | 0.004275 | 1 | 0.02451 | 1 |
| CNR1_HUMAN | 0.02413 | 2 | 0.04135 | 3 |
| NPTX2_HUMAN | 0.02577 | 3 | 0.09604 | 5 |
| NEC2_HUMAN | 0.03361 | 4 | 0.03407 | 2 |

**Table S5.** Correlation of CSF proteins with CSF tau (significant Pearson hits). Level of significance is indicated as *p*-value after Bonferroni correction.

| **BIOMARKER** | **PEARSON_P** | **PEARSON_RANK** | **SPEARMAN_P** | **SPEARMAN_RANK** |
| --- | --- | --- | --- | --- |
| BASP1_HUMAN | 3.518e-07 | 1 | 0 | 9 |
| 1433Z_HUMAN | 1.723e-06 | 2 | 0 | 15 |
| NEUM_HUMAN | 1.787e-06 | 3 | 0 | 6 |
| SMOC1_HUMAN | 2.127e-06 | 4 | 0 | 13 |
| KPYM_HUMAN | 3.523e-06 | 5 | 0 | 2 |
| FABPH_HUMAN | 4.606e-06 | 6 | 0 | 18 |
| NEDD8_HUMAN | 5.193e-06 | 7 | 0 | 3 |
| ALDOA_HUMAN | 6.519e-06 | 8 | 0 | 10 |
| NEUG_HUMAN | 6.767e-06 | 9 | 0 | 1 |
| TAGL3_HUMAN | 7.564e-06 | 10 | 0 | 7 |
| TYB10_HUMAN | 9.671e-06 | 11 | 0 | 5 |
| MDHC_HUMAN | 2.89e-05 | 12 | 0 | 8 |
| SORC1_HUMAN | 5.997e-05 | 13 | 0 | 19 |
| STMN1_HUMAN | 6.49e-05 | 14 | 0 | 4 |
| FOLR2_HUMAN | 6.922e-05 | 15 | 2.16e-05 | 89 |
| LFG2_HUMAN | 0.0001664 | 16 | 0 | 12 |
| AATC_HUMAN | 0.0001971 | 17 | 0 | 14 |
| PRRT2_HUMAN | 0.000644 | 18 | 0 | 11 |
| DYL2_HUMAN | 0.0008047 | 19 | 0 | 16 |
| SYUG_HUMAN | 0.001112 | 20 | 0 | 25 |
| 1433B_HUMAN | 0.001242 | 21 | 0 | 17 |
| SPON1_HUMAN | 0.001302 | 22 | 4.76e-07 | 37 |
| AT1A2_HUMAN | 0.001514 | 23 | 1.156e-05 | 72 |
| EPHB6_HUMAN | 0.001688 | 24 | 6.725e-07 | 39 |
| UBB_HUMAN | 0.001857 | 25 | 0 | 24 |
| SODC_HUMAN | 0.001913 | 26 | 0 | 23 |
| PALM_HUMAN | 0.00237 | 27 | 7.148e-08 | 30 |
| PGAM1_HUMAN | 0.002883 | 28 | 4.169e-07 | 35 |
| VTM2A_HUMAN | 0.003168 | 29 | 7.693e-09 | 29 |
| MARCS_HUMAN | 0.004051 | 30 | 1.547e-06 | 43 |
| TYB4_HUMAN | 0.004665 | 31 | 0 | 26 |
| ENPP2_HUMAN | 0.004919 | 32 | 0.02484 | 370 |
| HSP7C_HUMAN | 0.005774 | 33 | 0 | 28 |
| CMGA_HUMAN | 0.006569 | 34 | 1.044e-05 | 68 |
| HTRA1_HUMAN | 0.006834 | 35 | 0 | 22 |
| SZT2_HUMAN | 0.00781 | 36 | 0 | 27 |
| AT1A3_HUMAN | 0.008365 | 37 | 1.222e-06 | 41 |
| PEBP1_HUMAN | 0.01095 | 38 | 0 | 20 |
| ISLR2_HUMAN | 0.01149 | 39 | 0.0003508 | 186 |
| PITH1_HUMAN | 0.01362 | 40 | 1.02e-07 | 31 |
| SUSD5_HUMAN | 0.0146 | 41 | 2.355e-06 | 47 |
| CAD13_HUMAN | 0.01781 | 42 | 7.206e-07 | 40 |
| THIO_HUMAN | 0.02346 | 43 | 5.985e-07 | 38 |
| LDHB_HUMAN | 0.02376 | 44 | 4.068e-06 | 53 |
| TMED4_HUMAN | 0.024 | 45 | 0.0004008 | 192 |
| SH3L1_HUMAN | 0.02794 | 46 | 4.527e-07 | 36 |
| CH3L1_HUMAN | 0.0302 | 47 | 2.828e-07 | 34 |
| LY6H_HUMAN | 0.03831 | 48 | 4.216e-05 | 104 |
| CADM2_HUMAN | 0.03964 | 49 | 1.292e-06 | 42 |
| CYC_HUMAN | 0.04168 | 50 | 0.0002144 | 168 |

**Table S6.** Correlation of CSF proteins with CSF P-tau 181 (significant Pearson hits). Level of significance is indicated as *p*-value after Bonferroni correction.

| **BIOMARKER** | **PEARSON_P** | **PEARSON_RANK** | **SPEARMAN_P** | **SPEARMAN_RANK** |
| --- | --- | --- | --- | --- |
| AT1A2_HUMAN | 1.86e-06 | 1 | 3.26e-06 | 61 |
| SMOC1_HUMAN | 1.415e-05 | 2 | 0 | 11 |
| BASP1_HUMAN | 4.954e-05 | 3 | 0 | 8 |
| KPYM_HUMAN | 5.956e-05 | 4 | 0 | 2 |
| TYB10_HUMAN | 5.997e-05 | 5 | 0 | 3 |
| NEUM_HUMAN | 0.0001203 | 6 | 0 | 9 |
| NEDD8_HUMAN | 0.0001796 | 7 | 0 | 5 |
| ALDOA_HUMAN | 0.0002707 | 8 | 0 | 12 |
| STMN1_HUMAN | 0.0003174 | 9 | 0 | 7 |
| 1433Z_HUMAN | 0.0003247 | 10 | 4.173e-07 | 40 |
| ADA10_HUMAN | 0.0005247 | 11 | 0.002227 | 291 |
| FABPH_HUMAN | 0.0007064 | 12 | 0 | 17 |
| TAGL3_HUMAN | 0.0008992 | 13 | 0 | 4 |
| LFG2_HUMAN | 0.001386 | 14 | 0 | 15 |
| AATC_HUMAN | 0.001583 | 15 | 0 | 13 |
| MDHC_HUMAN | 0.001825 | 16 | 0 | 10 |
| FOLR2_HUMAN | 0.00283 | 17 | 4.435e-05 | 126 |
| PRRT2_HUMAN | 0.002831 | 18 | 0 | 14 |
| DYL2_HUMAN | 0.00517 | 19 | 0 | 16 |
| EPHB6_HUMAN | 0.005438 | 20 | 0 | 35 |
| SZT2_HUMAN | 0.005999 | 21 | 0 | 18 |
| SODC_HUMAN | 0.006455 | 22 | 0 | 22 |
| VTM2A_HUMAN | 0.006552 | 23 | 0 | 24 |
| UBB_HUMAN | 0.009009 | 24 | 0 | 26 |
| 1433B_HUMAN | 0.009766 | 25 | 6.926e-09 | 37 |
| HTRA1_HUMAN | 0.01054 | 26 | 0 | 25 |
| SUSD5_HUMAN | 0.01128 | 27 | 1.659e-06 | 51 |
| SPON1_HUMAN | 0.0143 | 28 | 0 | 29 |
| CMGA_HUMAN | 0.01528 | 29 | 3.488e-06 | 62 |
| TYB4_HUMAN | 0.01588 | 30 | 0 | 19 |
| AT1A3_HUMAN | 0.0185 | 31 | 0 | 28 |
| CAD13_HUMAN | 0.01916 | 32 | 0 | 30 |
| CYTL1_HUMAN | 0.01993 | 33 | 0.03647 | 402 |
| SYUG_HUMAN | 0.02036 | 34 | 0 | 20 |
| PITH1_HUMAN | 0.02388 | 35 | 0 | 32 |
| PALM_HUMAN | 0.02709 | 36 | 0 | 27 |
| SLIK1_HUMAN | 0.03152 | 37 | 2.572e-05 | 102 |
| PEBP1_HUMAN | 0.03373 | 38 | 0 | 23 |
| HSP7C_HUMAN | 0.03394 | 39 | 0 | 36 |
| LY6H_HUMAN | 0.03709 | 40 | 6.587e-07 | 45 |
| SORC1_HUMAN | 0.03984 | 41 | 0 | 6 |
| CALM_HUMAN | 0.04174 | 42 | 7.877e-08 | 38 |
| CADM2_HUMAN | 0.04503 | 43 | 0 | 33 |
| PGAM1_HUMAN | 0.04761 | 44 | 2.9e-05 | 106 |
| DDAH1_HUMAN | 0.04881 | 45 | 0.06808 | 424 |
| NEUG_HUMAN | 0.04962 | 46 | 0 | 1 |

**Table S7.** **Group comparisons** of CSF protein measurements (*i.e.*, 541 CSF proteins), for AD (*i.e*., P-tau 181/Aβ1-42 > 0.0779) *versus* non-AD (*i.e*., P-tau 181/Aβ1-42 ≤ 0.0779) CSF biomarker profiles, in all subjects. Level of significance is indicated as *p*-value after correction for multiple testing using the Benjamini-Hochberg procedure. Twenty-two reported hits present FDR ≤ 5%.

| BIOMARKER | P | FDR | RANK |
| --- | --- | --- | --- |
| 1433Z_HUMAN | 6.46E-06 | 0.002558 | 1 |
| SMOC1_HUMAN | 9.46E-06 | 0.002558 | 2 |
| NEUM_HUMAN | 3.09E-05 | 0.005566 | 3 |
| MDHC_HUMAN | 4.38E-05 | 0.005897 | 4 |
| BASP1_HUMAN | 5.45E-05 | 0.005897 | 5 |
| KPYM_HUMAN | 6.67E-05 | 0.006017 | 6 |
| SZT2_HUMAN | 0.0001925 | 0.01303 | 7 |
| ALDOA_HUMAN | 0.0001926 | 0.01303 | 8 |
| NEDD8_HUMAN | 0.0003518 | 0.01801 | 9 |
| 1433B_HUMAN | 0.000358 | 0.01801 | 10 |
| FABPH_HUMAN | 0.0003802 | 0.01801 | 11 |
| MARCS_HUMAN | 0.0003994 | 0.01801 | 12 |
| AATC_HUMAN | 0.0005209 | 0.02168 | 13 |
| TYB10_HUMAN | 0.0005761 | 0.02226 | 14 |
| STMN1_HUMAN | 0.0006722 | 0.02424 | 15 |
| CYC_HUMAN | 0.0008057 | 0.02724 | 16 |
| SODC_HUMAN | 0.001083 | 0.03447 | 17 |
| UBB_HUMAN | 0.001204 | 0.03617 | 18 |
| TYB4_HUMAN | 0.001283 | 0.03654 | 19 |
| TAGL3_HUMAN | 0.00153 | 0.03945 | 20 |
| SYUG_HUMAN | 0.001531 | 0.03945 | 21 |
| CH3L1_HUMAN | 0.001703 | 0.04187 | 22 |

**Table S8.** **Group comparisons** of CSF protein measurements (*i.e.*, 541 CSF proteins), for AD (*i.e*., P-tau 181/Aβ1-42 > 0.0779) *versus* non-AD (*i.e*., P-tau 181/Aβ1-42 ≤ 0.0779) CSF biomarker profiles, in subjects with cognitive impairment. Level of significance is indicated as *p*-value after correction for multiple testing using the Benjamini-Hochberg procedure. Ten reported hits present FDR ≤ 5%.

| BIOMARKER | P | FDR | RANK |
| --- | --- | --- | --- |
| SMOC1_HUMAN | 5.17E-05 | 0.02796 | 1 |
| KPYM_HUMAN | 0.0001509 | 0.03465 | 2 |
| 1433Z_HUMAN | 0.0001953 | 0.03465 | 3 |
| MDHC_HUMAN | 0.0002562 | 0.03465 | 4 |
| NEUM_HUMAN | 0.0004319 | 0.0416 | 5 |
| MARCS_HUMAN | 0.0004735 | 0.0416 | 6 |
| ALDOA_HUMAN | 0.0006063 | 0.0416 | 7 |
| AATC_HUMAN | 0.000699 | 0.0416 | 8 |
| CYC_HUMAN | 0.0007669 | 0.0416 | 9 |
| BASP1_HUMAN | 0.000769 | 0.0416 | 10 |


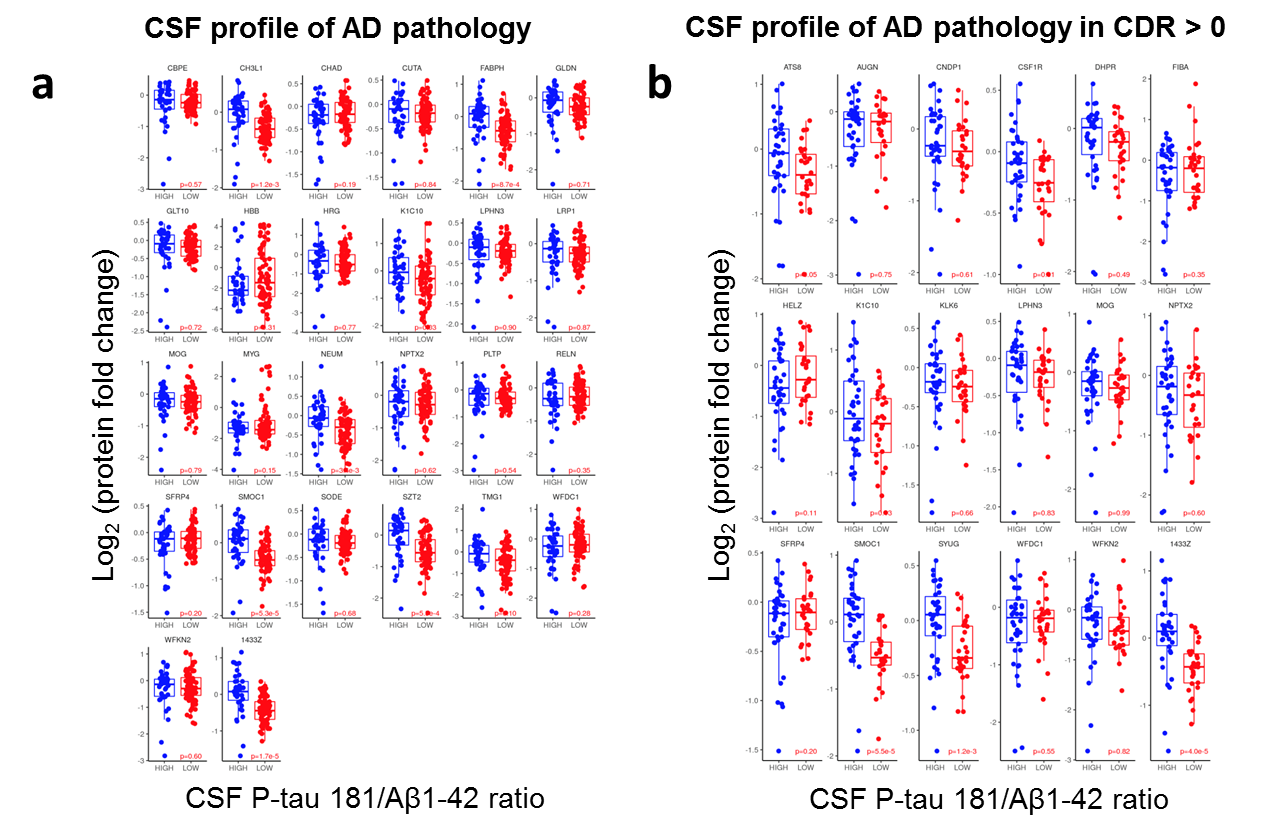


**Figure S1.** Box-plots of CSF proteins (previously selected with **LASSO** analyses*) according to CSF P-tau 181/Aβ1-42 ratio, *i.e.*, “high” when P-tau 181/Aβ1-42 > 0.0779 (blue dots) and “low” when P-tau 181/Aβ1-42 ≤ 0.0779 (red dots) for positive and negative CSF profiles of AD pathology, respectively, in all subjects (**a**) and restricted to subjects with cognitive impairment (*i.e.*, subset of individuals with CDR > 0) (**b**). Relative protein fold change ratios were used. Human proteins in the box-plots are given by their UniProtKB/Swiss-Prot entry name.

*CSF proteins selected with **LASSO** analysis for classification of non-AD *versus* AD CSF biomarker profiles (*i.e.*, P-tau 181/Aβ1-42 ≤ 0.0779 and P-tau 181/Aβ1-42 > 0.0779, respectively) in all subjects and in subjects with cognitive impairment are represented. The best classification model in the total sample included CBPE, CH3L1, CHAD, CUTA, FABPH, GLDN, GLT10, HBB, HRG, K1C10, LPHN3, LRP1, MOG, MYG, NEUM, NPTX2, PLTP, RELN, SFRP4, SMOC1, SODE, SZT2, TMG1, WFDC1, WFKN2, and 1433Z in addition of age and presence of the APOE ε4 allele while the reference model contains age and presence of the APOE ε4 allele. In cognitive impairment, the best classification model included ATS8, AUGN, CNDP1, CSF1R, DHPR, FIBA, HELZ, K1C10, KLK6, LPHN3, MOG, NPTX2, SFRP4, SMOC1, SYUG, WFDC1, WFKN2, and 1433Z in addition of gender and presence of the APOE ε4 allele while the reference model contains age, gender, years of education, and presence of the APOE ε4 allele. Human proteins listed above are given by their UniProtKB/Swiss-Prot entry name.


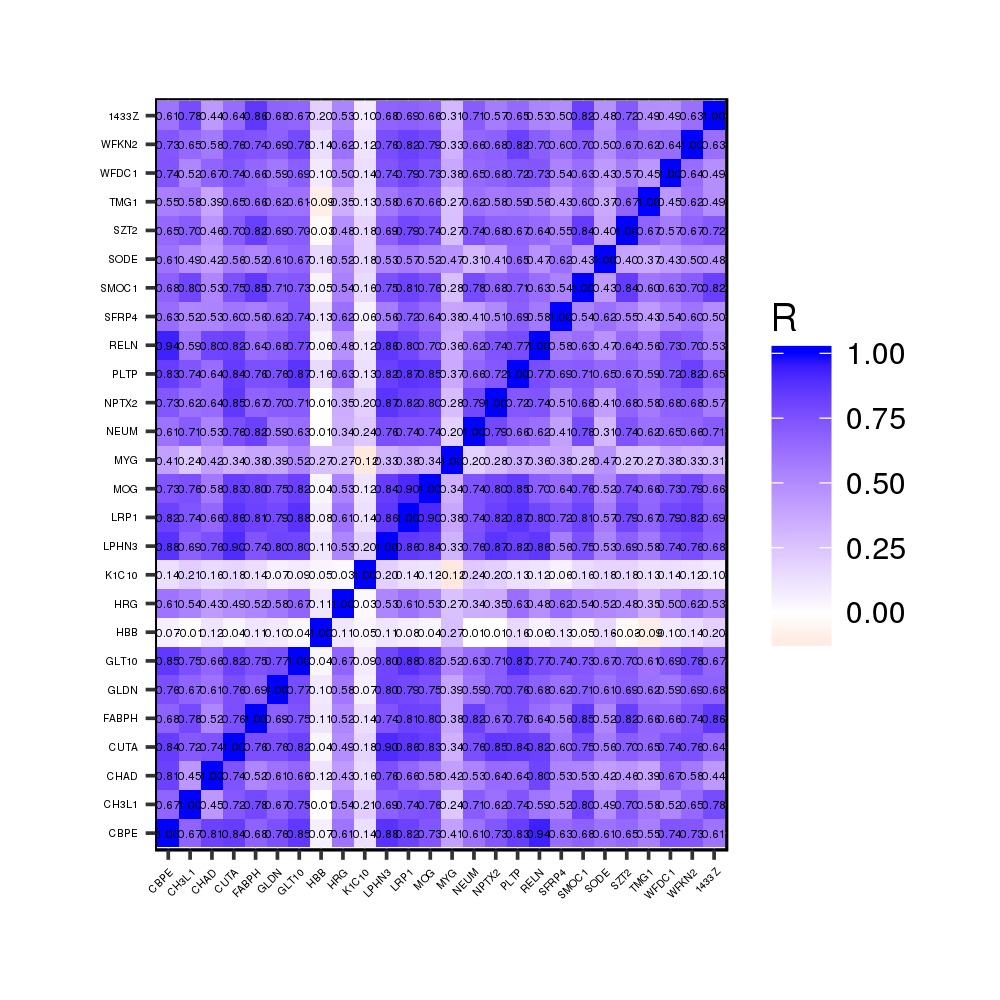


**Figure S2.** Pairwise correlation heatmap of the 26 CSF proteins, selected with **LASSO** analysis, for classification of non-AD *versus* AD CSF biomarker profiles (*i.e.*, P-tau 181/Aβ1-42 ≤ 0.0779 and P-tau 181/Aβ1-42 > 0.0779, respectively) for all subjects.


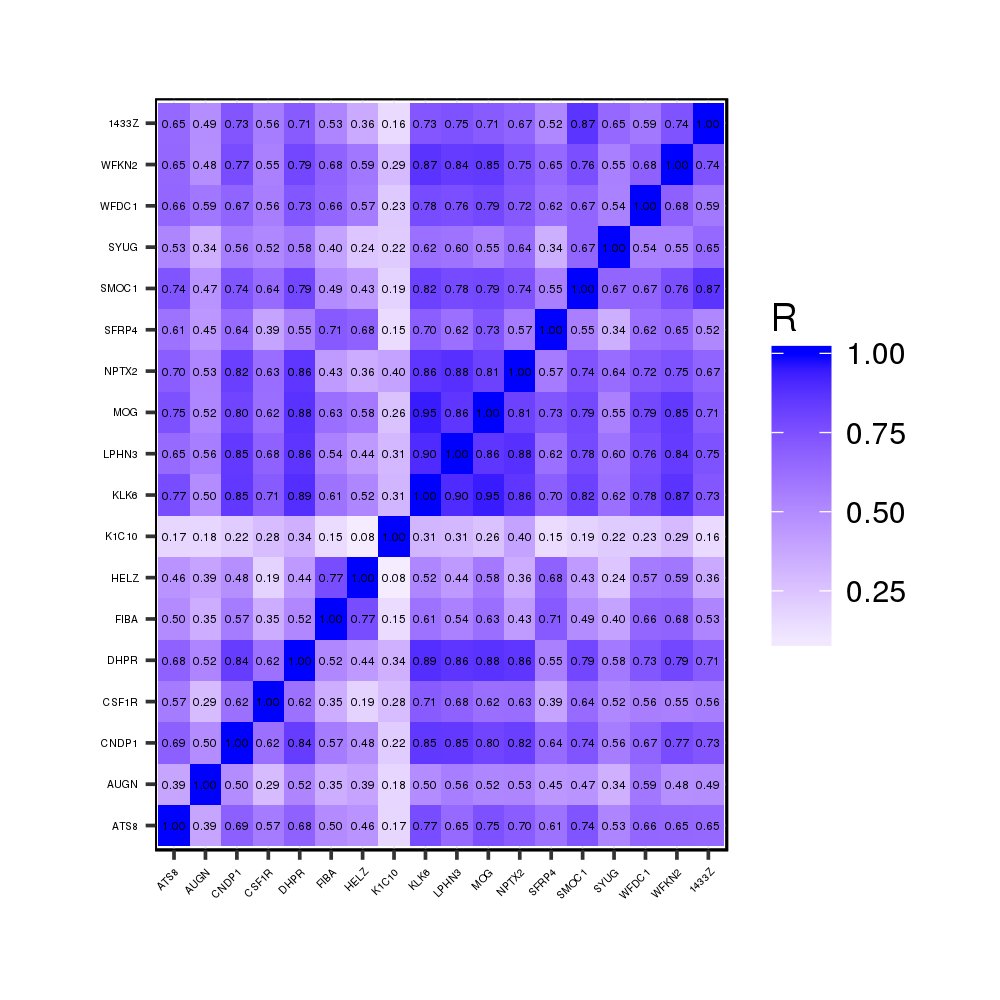


**Figure S3.** Pairwise correlation heatmap of the 18 CSF proteins, selected with **LASSO** analysis, for classification of non-AD *versus* AD CSF biomarker profiles (*i.e.*, P-tau 181/Aβ1-42 ≤ 0.0779 and P-tau 181/Aβ1-42 > 0.0779, respectively) for subjects with cognitive impairment.


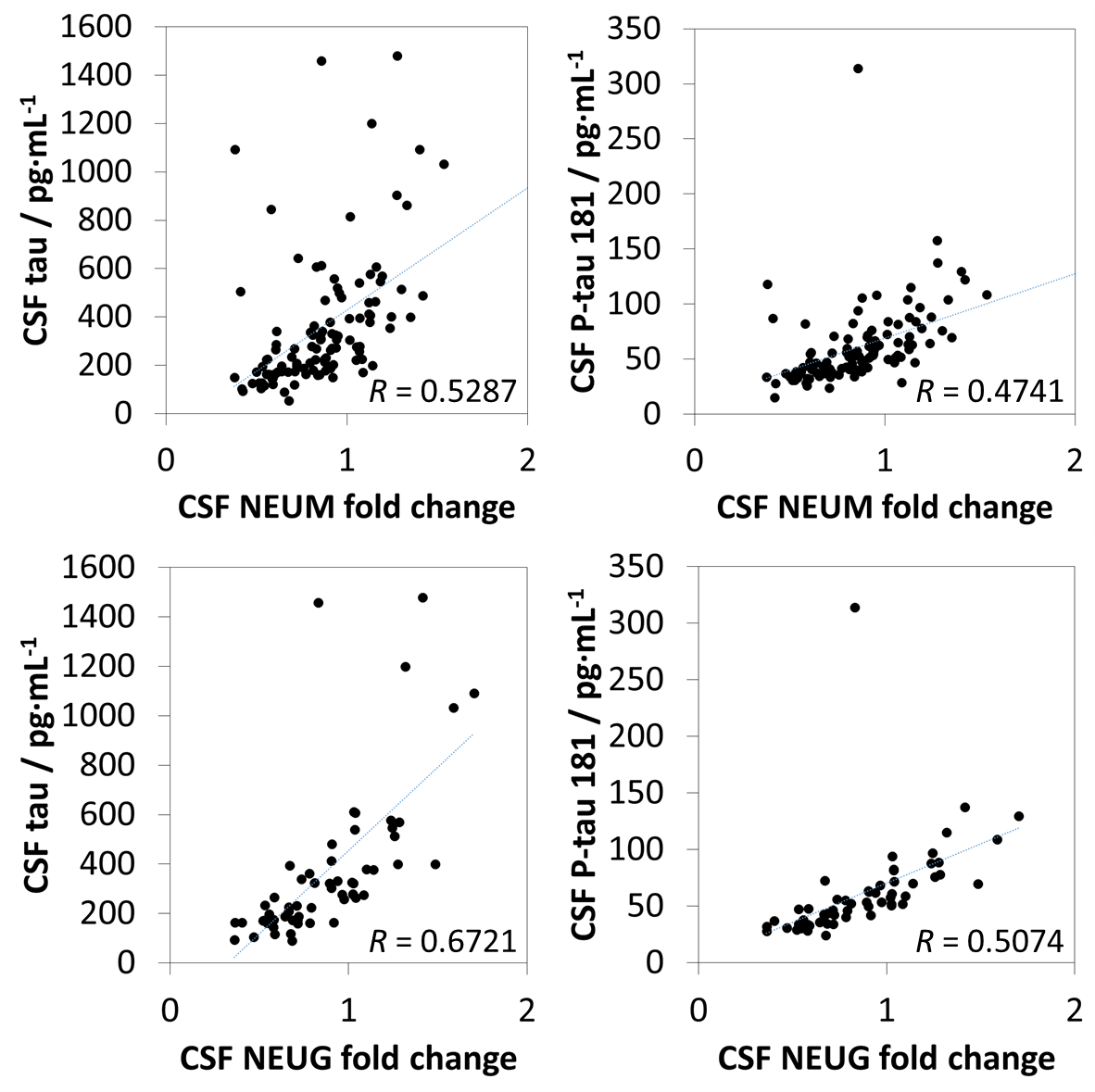


**Figure S4.** Correlations of CSF neurogranin (NEUG) and neuromodulin (NEUM) with CSF tau and P-tau 181.


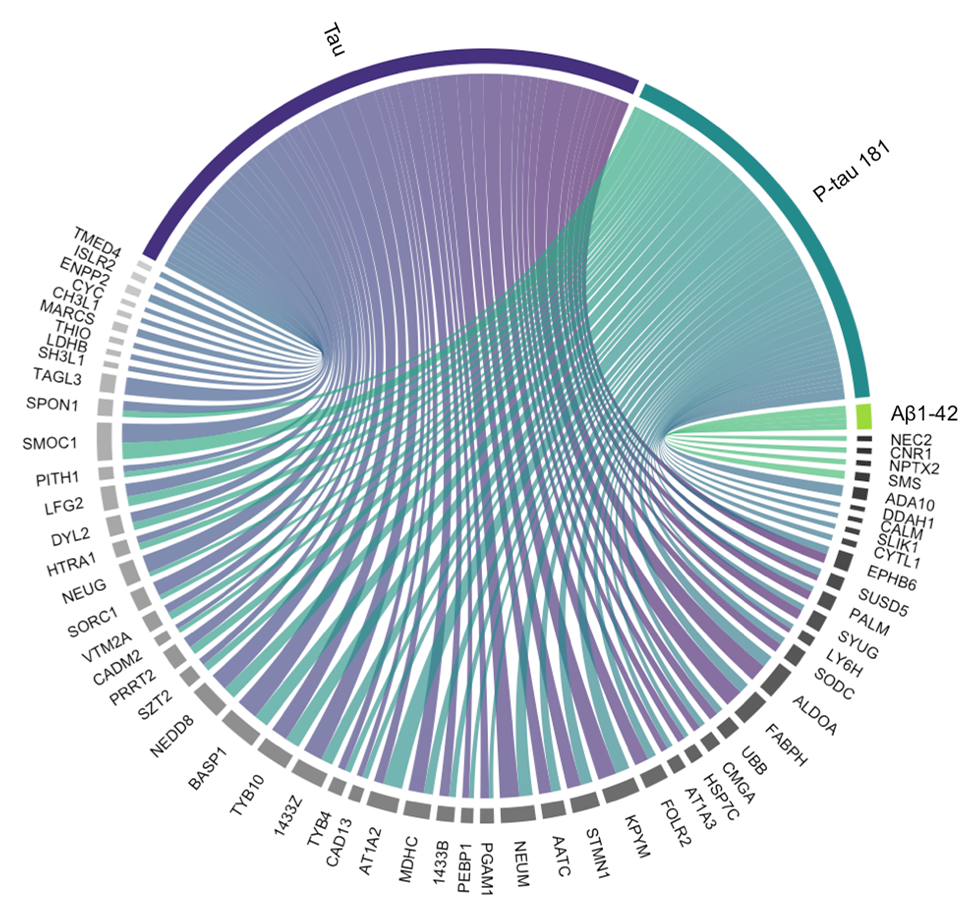


**Figure S5.** Chord diagram displaying the relationship of 59 CSF proteins with CSF tau, P-tau 181, and/or Aβ1-42. The thickness of the connecting arcs represents log_2_(1/*p*-value).

**
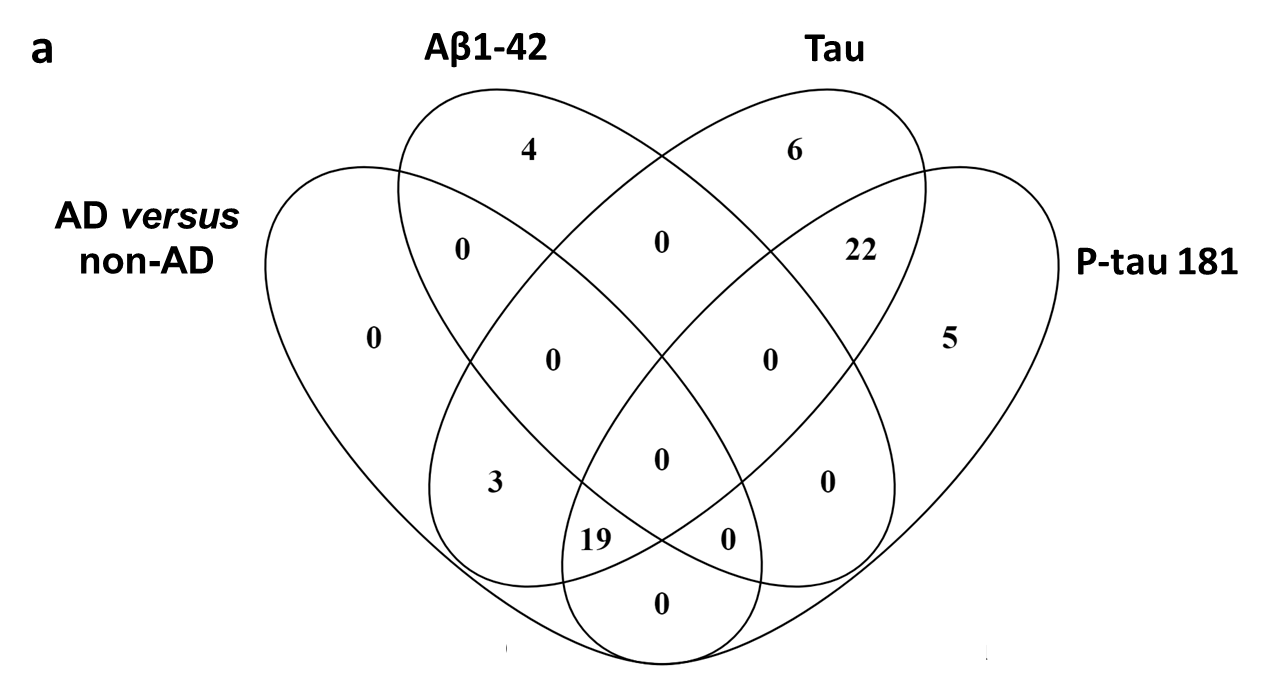
**

**
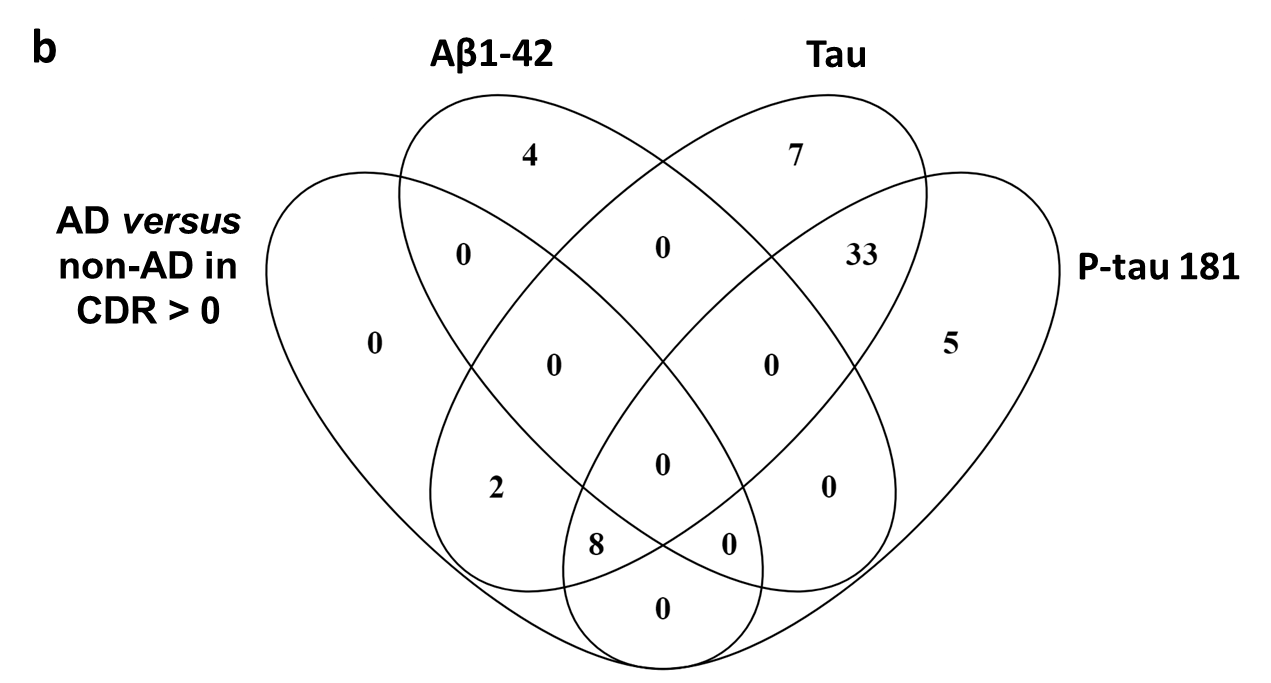
**

**Figure S6.** Venn diagram of CSF proteins with significant **group comparison** differences between AD *versus* non-AD CSF biomarker profiles (*i.e.*, P-tau 181/Aβ1-42 > 0.0779 and P-tau 181/Aβ1-42 ≤ 0.0779, respectively) in all subjects (22 proteins) and those correlating with CSF Aβ1-42 (four proteins), tau (50 proteins), and P-tau 181 (46 proteins) (**a**). Venn diagram of CSF proteins with significant **group comparison** differences between AD *versus* non-AD CSF biomarker profiles (*i.e.*, P-tau 181/Aβ1-42 > 0.0779 and P-tau 181/Aβ1-42 ≤ 0.0779, respectively) in subjects with cognitive impairment (10 proteins) and those correlating with CSF Aβ1-42 (four proteins), tau (50 proteins), and P-tau 181 (46 proteins) (**b**).


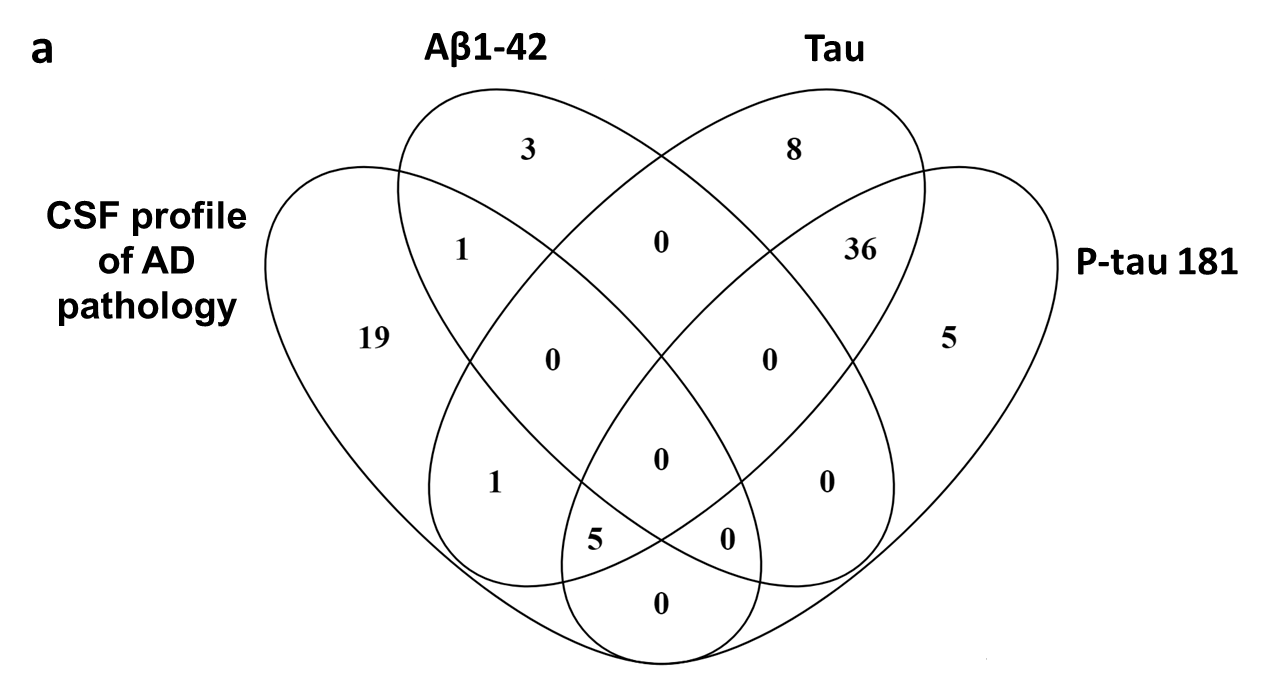


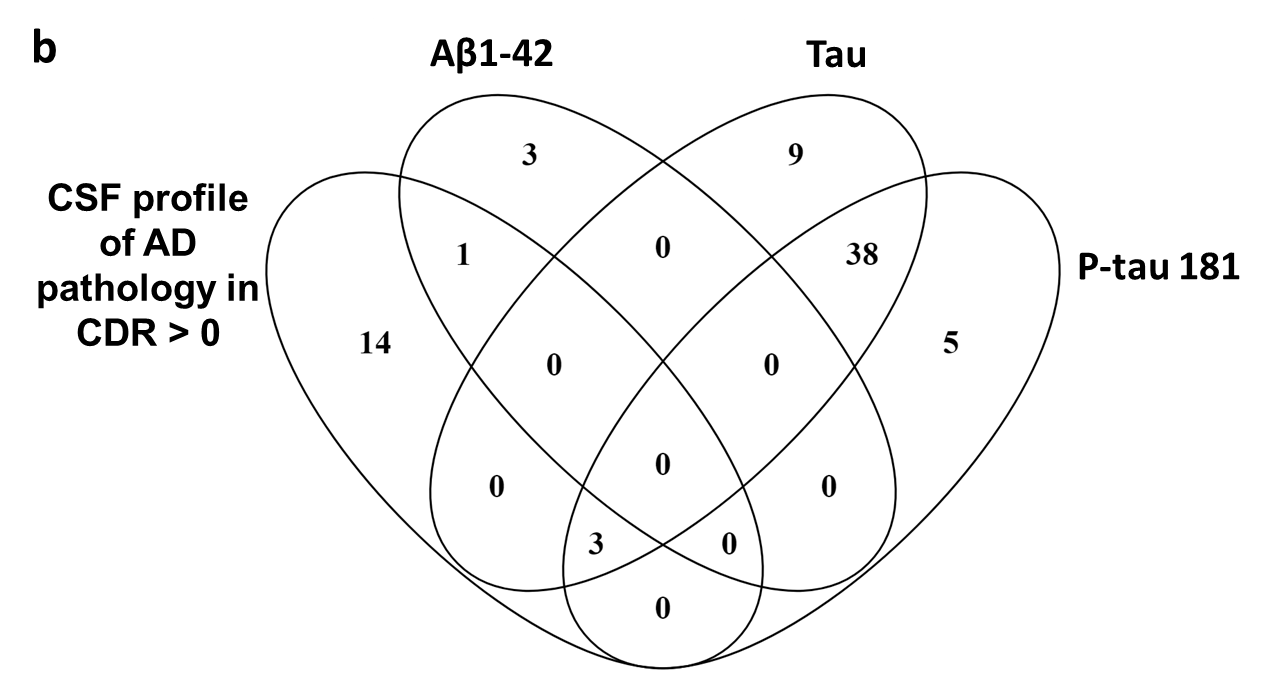


**Figure S7.** Venn diagram of CSF proteins selected with **LASSO** analysis and used for classification of non-AD *versus* AD CSF biomarker profiles (*i.e.*, P-tau 181/Aβ1-42 ≤ 0.0779 and P-tau 181/Aβ1-42 > 0.0779, respectively) in all subjects (26 proteins) and those correlating with CSF Aβ1-42 (four proteins), tau (50 proteins), and P-tau 181 (46 proteins) (**a**). Venn diagram of CSF proteins selected with **LASSO** analysis and used for classification of non-AD *versus* AD CSF biomarker profiles (*i.e.*, P-tau 181/Aβ1-42 ≤ 0.0779 and P-tau 181/Aβ1-42 > 0.0779, respectively) in subjects with cognitive impairment (18 proteins) and those correlating with CSF Aβ1-42 (four proteins), tau (50 proteins), and P-tau 181 (46 proteins) (**b**).
